# Integrating Hyperuniform Disordered Light Trapping Layers in Ultrathin c‑Si Solar Cells

**DOI:** 10.1021/acsphotonics.6c01224

**Published:** 2026-10-12

**Authors:** Alexander Lambertz, Nayeun Lee, Ali P. Vafa, Viktoryia Shautsova, Fenghao Xu, Jung-Hwan Song, Nicholas A. Güsken, Skyler Selvin, Marian Florescu, Mark L. Brongersma, Jorik van de Groep, Esther Alarcon-Llado

**Affiliations:** † 55952 NWO-I Institute AMOLF, Science Park 104, 1098XG Amsterdam, The Netherlands; ‡ Van der Waals–Zeeman Institute, Institute of Physics, 1234 University of Amsterdam, Science Park 904, 1098XH Amsterdam, The Netherlands; § Department of Materials Science and Engineering, 6429 Stanford University, 496 Lomita Mall, Stanford, California 94305, United States; ∥ Optoelectronics Research Centre, 7423 University of Southampton, Southampton SO17 1BJ, U.K.; ⊥ Institute for Photonic Quantum Systems (PhoQS), Paderborn University, Warburger Str. 100, 33098 Paderborn, Germany; # Van’t Hoff Institute of Molecular Sciences, University of Amsterdam, Science Park 904, 1098XH Amsterdam, The Netherlands

**Keywords:** ultrathin photovoltaics, light trapping, hyperuniformity, hyperuniform correlated disorder, c-Si

## Abstract

Ultrathin crystalline silicon solar cells require advanced light trapping to compensate for weak near-infrared (NIR) absorption. Here, we integrate hyperuniform disordered light-engineered (HUDLE) nanopatterns with high-quality surface passivation in 4.8 μm c Si cells and demonstrate robust device operation enabled by broadband coupling to guided modes. HUDLE layers enhance the fraction of power scattered beyond the escape cone by up to 30% in the NIR and increase average external quantum efficiency (EQE) by >50% over flat cells and 10% over periodic designs from 750 to 1000 nm. We directly compare periodic and hyperuniform nanopatterns and show that the continuous spatial frequency distribution of HUDLE leads to more homogeneous coupling of light into guided modes. Importantly, this optical enhancement is achieved without compromising open-circuit voltage or fill factor, demonstrating compatibility between advanced nanophotonics and high electronic quality in ultrathin devices. These results establish hyperuniform disorder as a powerful photonic design strategy for ultrathin silicon photovoltaics.

## Graphical abstract

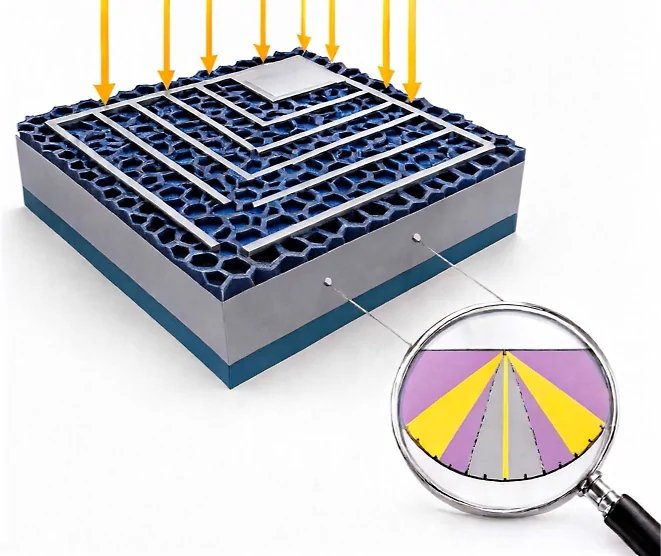

## Introduction

The increasing global demand for sustainable energy sources has driven the development of solar cell technologies, with crystalline silicon (c-Si) solar cells dominating the photovoltaic market.^1^ Despite their dominance, conventional c-Si solar cells face limitations in terms of weight, cost, and flexibility, particularly for emerging applications such as building-integrated photovoltaics and in the mobility sector.^2^ A promising approach to overcome these limitations is to reduce the thickness of c-Si absorbers to the micrometer scale.^3,4^ However, c-Si solar cells with absorber thickness below 10 μm experience severe optical losses,^3^ as conventional light-management strategies—such as micron-scale pyramidal texturing—are incompatible with such thin devices.^5,6^ To compensate, advanced nanoscale light-trapping schemes must enhance absorption without degrading electronic quality. At these thicknesses, surface recombination becomes a dominant source of dark current, and any increase in surface area from nanostructuring proportionally intensifies recombination losses, resulting in reduced open-circuit voltage and fill factor.^7–9^ This creates a stringent trade-off between optical enhancement and electronic performance.

Recent advancements in nanofabrication and light trapping strategies have shown promise in improving the performance of ultrathin Si solar cells.^3,10^ Pattern design strategies to enhance light absorption include periodic arrays of inverted nanopyramids^11–13^ and nanocones,^14^ random patterns through deep reactive ion etching^15^ or using a random nanoparticle mask,^16,17^ multiresonant periodic arrays,^18–20^ and engineered random patterns.^21,22^ Control over light scattering in these structures is afforded by diffraction orders of the periodic arrays,^13^ localized Mie resonances,^23^ or a combination of both.^18,24,25^ Building on these advances, power conversion efficiencies up to 13.7%^14^ and 15.7%^11^ have been achieved with solar cells of only 10 μm thickness.

Achieving high efficiencies with even thinner absorber layers requires scattering patterns that are tailored to the wafer thickness. Here, the scattering properties of engineered random light trapping structures with hyperuniform disorder (HUD)^26^ present a compelling opportunity: the placement of Mie-like scatterers in a mutual spatial arrangement that exhibits long-range uniformity yet no sharp diffraction peaks. Instead, the ensemble features a bounded but isotropic and continuous interval of spatial frequencies, thereby combining advantages of periodic and random patterns instead of sacrificing one desirable property for the other.^27–29^

This work experimentally validates broadband guided-mode coupling in functioning ultrathin solar cell devices by integrating Hyperuniform Disordered Light Engineered (HUDLE) scattering patterns with 4.8 μm thick c-Si absorbers. Our device architecture intentionally omits a metallic back reflector, thereby providing a clean platform to directly probe the front-surface scattering efficiency of the HUDLE layer into large in-plane momenta. While the strong scattering capabilities of HUDLE layers were previously demonstrated through enhanced absorption in 1 μm thick c-Si slabs,^30^ here we show that the broad scattering frequency band of the HUDLE results in more efficient near-infrared photocurrent generation compared to periodic patterns. Using spectrally resolved external quantum efficiency measurements in combination with wave-optics simulations, we directly correlate the structure factor of hyperuniform patterns with their momentum-resolved scattering response in a functioning ultrathin device. We show that the continuous spatial frequency band of HUDLE textures enables broadband coupling of near-infrared photons into guided modes, in contrast to the discrete diffraction channels of periodic lattices. Importantly, despite the increased surface area introduced by HUDLE nanostructuring, we experimentally demonstrate that the open-circuit voltage and fill factor remain uncompromised, confirming that the intricate hyperuniform textures are compatible with high-quality surface passivation in ultrathin architectures.

Together, these results establish hyperuniform disorder as a powerful framework for engineering light–matter interactions in optically thin semiconductor slabs.

## Results and Discussion

### Integration of Hyperuniform Nanopatterns into Ultrathin Devices

To experimentally probe enhanced optical absorption in ultrathin light absorbers, we integrate hyperuniform disordered light engineered (HUDLE) nanopatterns into 4.8 μm thick c-Si solar cells fabricated on silicon-on-insulator substrates (Figure 1a). The absence of a metallic back reflector in this architecture ensures that light trapping arises primarily from front-surface scattering, providing a controlled platform to directly evaluate coupling into large in-plane wave vectors beyond the escape cone (Figure 1b). By comparing flat, periodic, and hyperuniform nanopatterns fabricated on the same wafer, we isolate the role of the spatial frequency distribution in governing guided-mode excitation and photocurrent generation.

**Figure 1:**
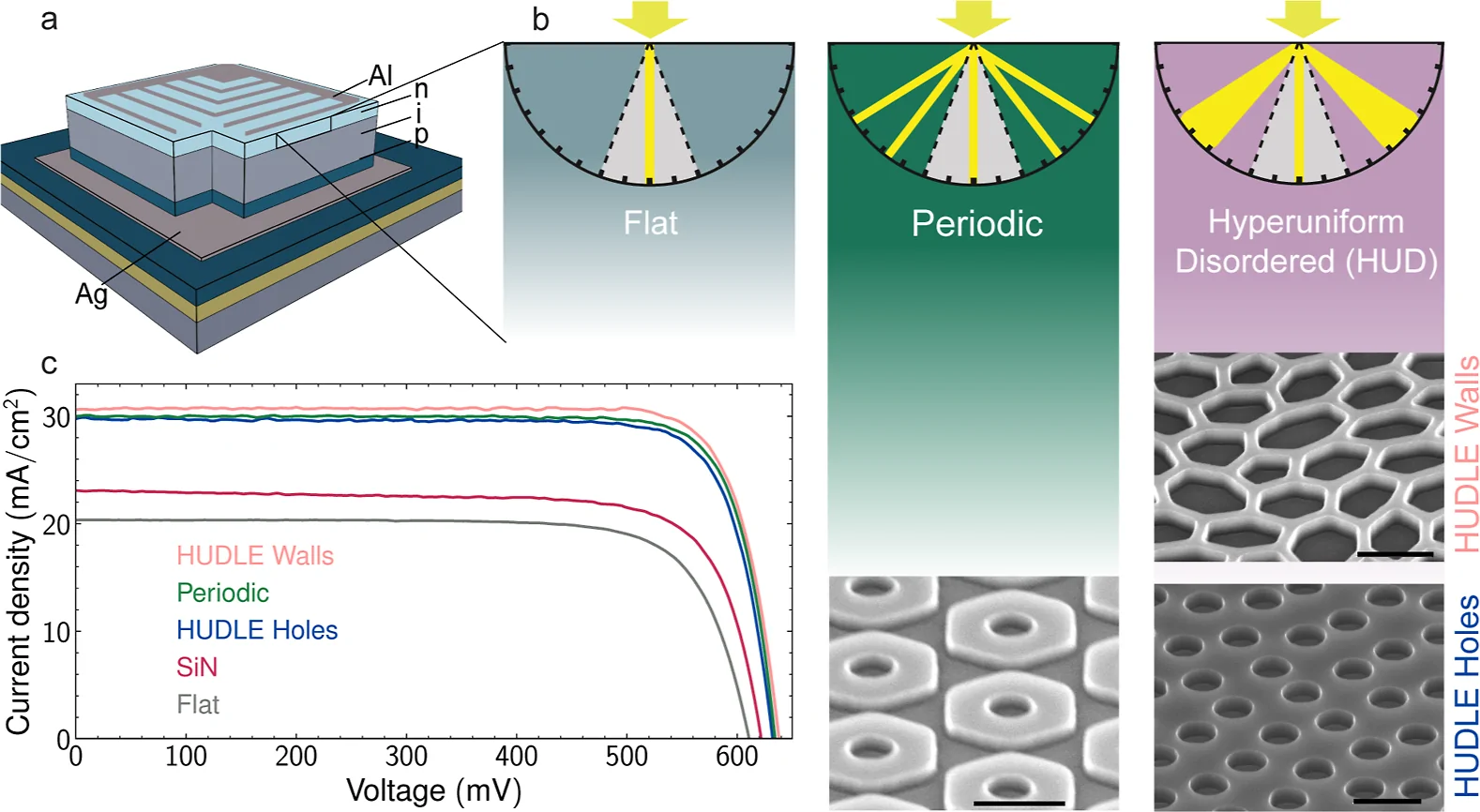
Schematic, operation principle, and performance metrics of 4.8 μm thick c-Si solar cell devices (a) Sketch of the 4.8 μm ultrathin solar cell device grown on a silicon-on-insulator (SOI) wafer with an initial seed layer thickness of 2 μm , on top of a 500 nm buried silicon dioxide layer separating the device from the 500 μm thick Si handle wafer. (b) Illustrative representation of light scattering and diffraction through different surface morphologies: flat (left), periodic nanopattern (middle), and hyperuniform disordered nanopattern (HUDLE, right). The corresponding scattering patterns into the substrate are highlighted as yellow lobes in the polar plots where the gray shaded area indicates the escape cone of light. Scanning electron micrographs of periodic and two HUDLE designs are also shown (scale bars: 500 nm). (c) Current–voltage characteristics obtained at 1-sun illumination for the best performing solar cell of each type.

The spatial frequencies in both the HUDLE and periodic nanopatterns were carefully designed to maximize the absorption of the solar spectrum. Arrays of subwavelength structures can scatter and diffract incident light into the absorbing layer beyond the escape cone of light (i.e., at angles beyond the critical angle).^31^ This leads to total internal reflection and thereby light trapping if the in-plane momentum of scattered light matches those of the guided modes of the thin-film absorber.^32^ Crucially, the distribution of the scattered light across the range of wave vectors directly dictates the (subset of) guided modes into which light is coupled. Here, guided modes are standing-wave solutions to Maxwell’s equations in the absorbing layer that enable in-plane propagation, thereby strongly increasing the optical path length. This is essential for ultrathin devices where a wide range of photon energies are not fully absorbed within a single-pass through the absorbing layer, since a 5 μm thick c-Si film only absorbs <50% at wavelengths λ ≳ 750 nm (see the Supporting Information). Therefore, light trapping is particularly crucial for near-infrared (NIR) photons.

To illustrate this, Figure 1b shows the angular distribution of light scattered off the (patterned) air-silicon interface. Light entering silicon through a flat surface, i.e., without nanostructuring, (left) has in-plane momenta exclusively within the escape cone. As such, light trapping cannot occur. Applying a periodic structuring with a periodicity *p* on the order of the wavelength of light (middle) introduces diffractive orders at distinct in-plane momenta $k_{∥}=\sqrt{{\left(2\pi m/p\right)}^{2}+{\left(2\pi n/p\right)}^{2}}$ , where the integers *m*, *n* ∈ {0, ±1, ±2, ..} represent diffraction orders along the mutually orthogonal 2D lattice directions. The angles associated with these *k*_∥_ can lie beyond the escape cone and result in light trapping, but only at discrete wavelengths where *k*_∥_ matches one of the guided modes.^33^ In contrast, the continuous spatial frequencies in hyperuniform disordered nanopatterned surfaces (right) offer a continuous range *k*_∥_ ∈ [10, 20] μm^–1^, which falls within the momentum range of the guided modes at the edge of the light line (see Figure S2). This in principle facilitates optimal access to guided modes in the absorber over a broad spectral range, while minimizing reflection losses within the escape cone.

The engineered nanopatterns are transferred into functioning devices by etching approximately 245 nm into the top layer of the solar cell stack. The nanopattern depth was targeted at 255 nm, based on a representative numerical optimization showing a broad optimum around 250–300 nm (see Supporting Information, Figure S3). Due to fabrication tolerances, the fabricated structures had a final depth of approximately 245 nm. To enable statistically meaningful comparisons, large-area (cm scale) fields of each design (two HUDLE designs, one periodic reference and one unpatterned) are defined on the same wafer. After etching, we passivate the front surface by subjecting the wafer to dry thermal oxidation to form a layer of silicon dioxide. Part of the unpatterned wafer is also subjected to SiN_*x*_ deposition, to improve antireflectance. Individual 1.6 × 1.6 mm^2^ devices are subsequently formed by mesa isolation and contact deposition. Note that the p-type contact is not formed as a full-area back electrode but is instead contacted laterally at the mesa sidewalls, such that no reflective metallic layer is present beneath the active area, and thus ensuring that optical enhancement originates from front-surface scattering rather than cavity effects (see a device sketch Figure S4). This fabrication approach yields ∼25 of nominally identical devices per pattern type, all processed and passivated simultaneously on the same wafer. As a result, differences in performance can be directly attributed to the nanopattern design rather than variations in material quality or processing conditions.

The current–voltage (*J*–*V*) characteristics of the best-performing cells of each pattern type under AM1.5G illumination are shown in Figure 1c, with the corresponding photovoltaic parameters summarized in the Table in Figure 2. As a baseline, the flat cell with SiO_2_ passivation already reaches ∼90% of the single-pass absorption limit of 4.8 μm Si (Figure S1). Adding a SiN_*x*_ antireflection layer further increases the short-circuit current density by ∼3 mA/cm^2^, yielding *J*_SC_ = 23.1 mA/cm^2^, close to the double-pass absorption limit of the SOI stack, i.e. with the oxide back-reflecor (∼25 mA/cm^2^). This confirms the excellent electronic quality of the p–i–n junction and charge collection. The high *V*_OC_ of the unpatterned cells further indicates effective surface passivation, which is particularly critical for ultrathin absorbers where surface recombination dominates.^34,35^

Introducing nanopatterns at the front surface substantially enhances device performance through combined antireflection and light-trapping effects. The structured cells exhibit up to a 50% relative increase in *J*_SC_ compared to the flat reference. In addition, all patterned devices maintain fill factors above 80% and show consistently high *V*_OC_ values, indicating that any recombination losses introduced by the increased surface area remain limited. At the same time, the SiN_*x*_-covered flat cells show a small reduction in their fill factor, despite increasing their *J*_SC_ through improved antireflection, which we therefore attribute to unintended defect formation induced by the SiN_*x*_ deposition process. Dark *J*–*V* analysis using the two-diode model^36^ (Figure S5) reveals comparable recombination parameters (*J*_01_) for flat and patterned cells, confirming preserved surface passivation quality. This demonstrates that hyperuniform nanopatterning can be integrated into ultrathin silicon solar cells without measurable degradation of the key electrical performance metrics.

We note that the best-performing device, incorporating a HUD wall network, reaches a very high power conversion efficiency of (16.2 ± 0.5)% (see Table 1 in Figure 2). This value is derived from unmasked measurements, and therefore likely overestimated and not intended as a certified record (see the discussion on the active area definition in the Supporting Information). Yet, all device types were fabricated on the same wafer and characterized under identical conditions. The relative efficiency trends therefore primarily reflect differences between nanopattern designs, while the absolute efficiency values should be interpreted with the active-area and illumination-geometry considerations discussed in the Supporting Information. Figure 2 (bottom panel) shows the statistical distribution of efficiencies measured across multiple devices fabricated on the same wafer. The median efficiencies confirm the systematic improvement for the nanopatterned designs, particularly in the HUD wall network. The small device-to-device variation highlights the robustness of the platform and enables statistically meaningful comparison of the different nanopattern geometries.

**Figure 2:**
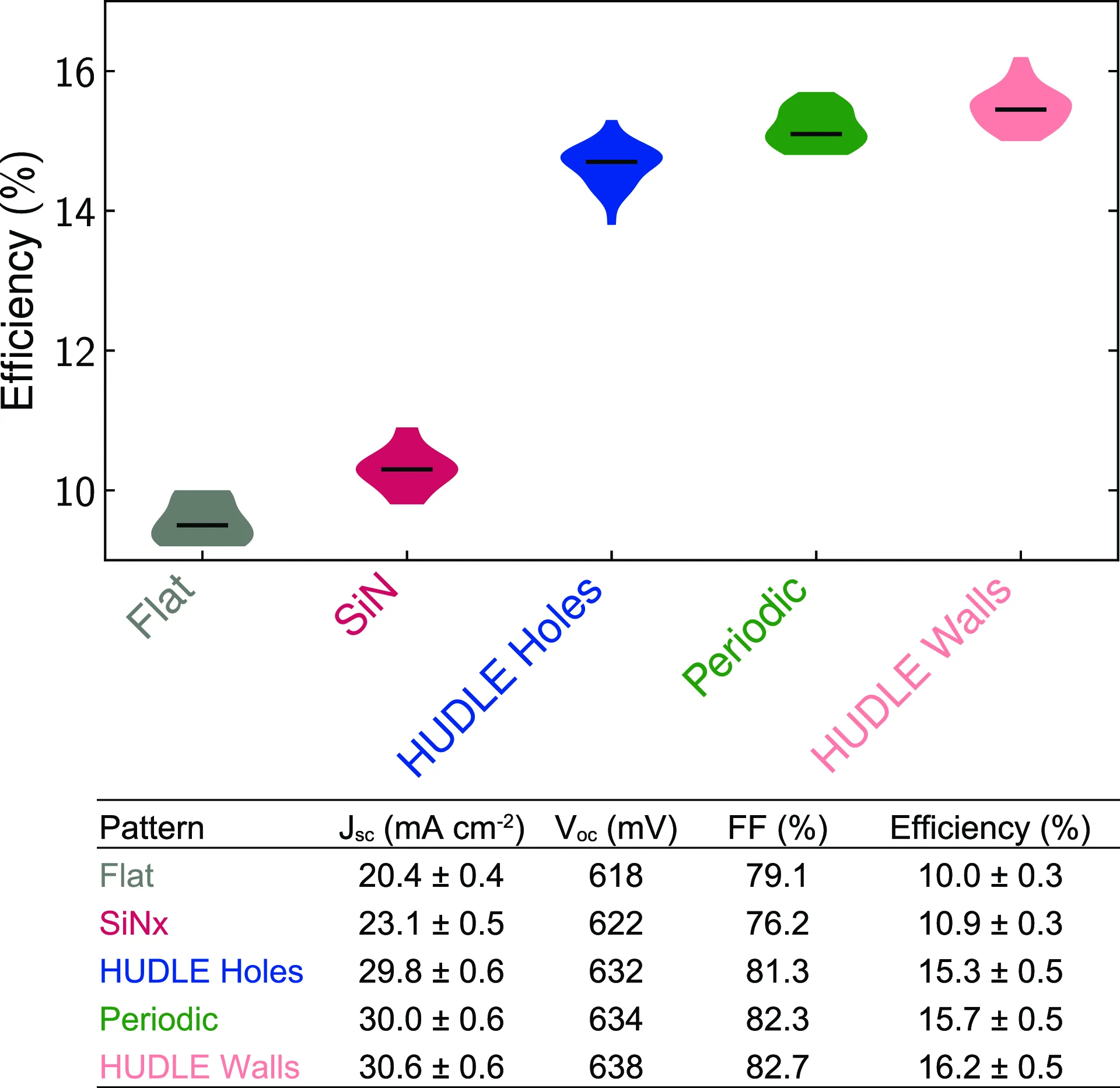
Photovoltaic parameters and efficiency statistics (top panel) table of performance parameters for the best solar cells of each type. (Bottom panel) statistical spread and efficiency comparison of the same set of devices (*N* = 25 for patterned/flat, *N* = 9 for SiN_*x*_), demonstrating reproducible performance across nanopattern types. Here, *N* indicates the number of identical cells that were characterized for each pattern type.

### Spectrally Resolved Characterization

While the current–voltage characteristics establish overall performance differences between the nanopattern designs, they do not reveal the spectral origin of these enhancements. To do so, we perform spectrally resolved external quantum efficiency (EQE) measurements for all device types (Figure 3a) and compare them to numerically simulated absorptance in the device stack (Figure 3b). The shaded regions around the EQE data visualize the uncertainty of the measurements, which are dominated by fluctuations of the incoming laser power. The gray shaded region from 670 to 700 nm indicates the spectral region that is inaccessible with our experimental setup. The optical simulations were done using rigorous coupled-wave analysis (RCWA), as described in the Methods section.

**Figure 3:**
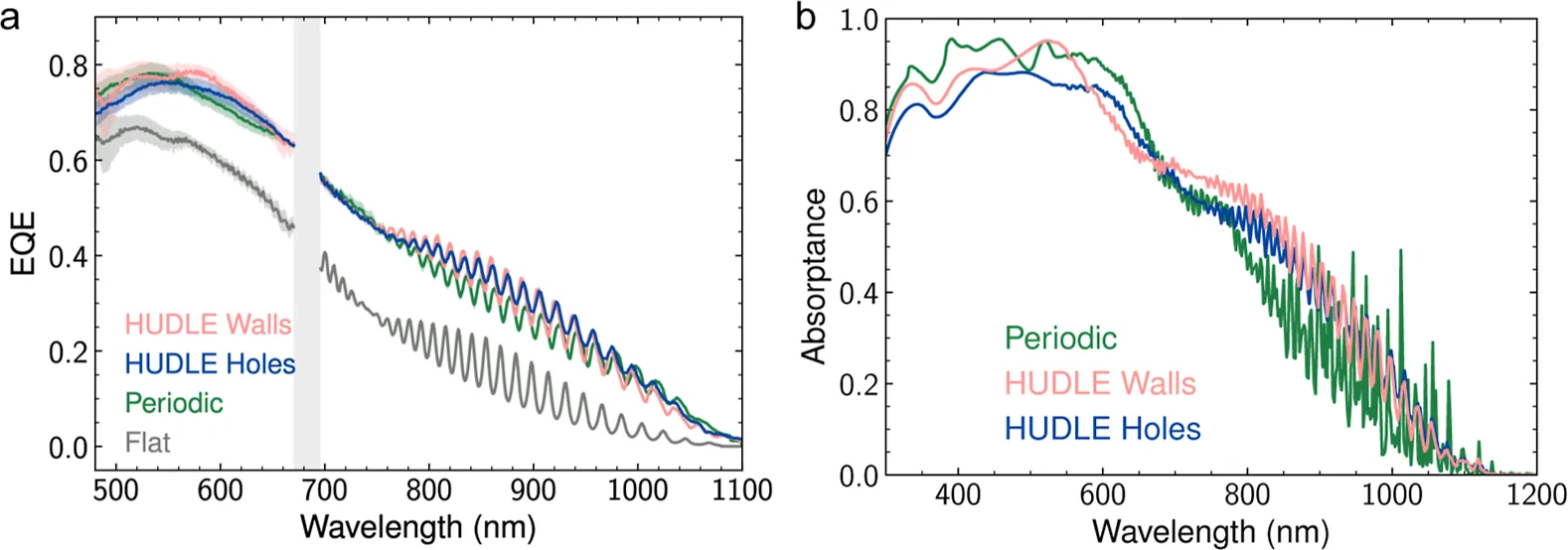
Spectral optoelectronic analysis. (a) Measured external quantum efficiency spectra of flat and patterned cells. The shaded regions around the curves indicates measurement uncertainty due to laser fluctuations, while the gray spectral region highlights the wavelength range that is inaccessible to our experimental setup. (b) Simulated absorptance of the corresponding device stacks.

To interpret the spectral response, we distinguish between two wavelength regimes: λ < 600 nm and λ > 800 nm. At short wavelengths, absorption in silicon is sufficiently strong that most photons are absorbed within a single pass through the 4.8 μm thick device. As a result, the EQE in this spectral region predominantly probes the antireflective (AR) performance of the front surface and internal quantum efficiency (IQE) rather than any absorption enhancement from light trapping.

The presence of nanopatterning clearly improves the EQE compared to the flat reference. All three types of nanopatterned devices show a very similar EQE at short wavelengths. From the previous section, we do not expect major effects of the nanopatterning on the electronic quality of the devices. Therefore, the improved EQE in this spectral region is most likely due to the improved AR in the patterned surfaces, where the combination of air and silicon lowers the effective refractive index of the patterned layer and thereby reduces the index contrast at the air–silicon interface. Considering the air filling fraction values (Periodic: 46.6%; HUDLE Walls: 45.7%; HUDLE Holes: 31.7%), we would expect the strongest AR in the periodic pattern, for which the EQE is marginally higher. This is confirmed by the simulations in Figure 3b, where simulated absorptance in the device stack at short wavelengths follows the trend in air filling fraction (i.e., Periodic > HUDLE Walls > HUDLE Holes).

On the other hand, the light is not fully absorbed within the device stack at longer wavelengths (λ > 800 nm). In this regime, all EQE spectra exhibit Fabry–Pérot (FP) oscillations, consistent with the free spectral range expected from the 4.8 μm device thickness together with the additional 2 μm silicon seed layer. These high-frequency oscillations are further modulated by lower frequency FP features arising from the 500 nm buried SiO_2_ layer.

The amplitude of the FP oscillations is markedly reduced in the presence of nanopatterns. This suppression indicates that a substantial fraction of the incident light is redistributed into large in-plane momenta beyond the escape cone, diminishing the coherent back-reflection within the stack. The resulting damping of the interference fringes provides a first indirect signature of enhanced light trapping by the nanopatterns. This picture is confirmed by the simulated absorptance: among the textured devices, the two HUDLE patterns show the highest absorptance across most of the NIR, precisely the range where the intrinsic absorption of ultrathin Si is the weakest and light trapping is most needed. The same trend appears in the measured EQE, which is enhanced over the entire NIR for both HUDLE devices relative to that of the periodic and flat references. This close correspondence between measured EQE and simulated absorptance confirms that the improved response originates from enhanced light trapping by the correlated disorder texture, rather than from differences in carrier collection between the devices.

In the following, we examine the origin of this improved light trapping by investigating the light-scattering properties of HUDLE layers.

### Hyperuniform Light Trapping Mechanism

Generally speaking, efficient light trapping requires suppressed scattering within the escape cone (*k*_∥_ < *k*_*c*_), while enhancing redistribution of light into large *k*_∥_ to couple to guided modes. In a weak-scattering picture, the momentum distribution of scattered light is set by the interplay between the individual scatterer response, or form factor, and the spatial correlations of the scatterer arrangement, encoded in the structure factor. For the present high-index resonant interface, the power spectral density (PSD) provides a useful design metric for the available spatial frequencies, while full-wave simulations are required to quantify the actual scattering response.

HUDLE patterns are engineered such that their structure factor vanishes near *k*_∥_ = 0 (similar to periodic patterns) and exhibits a continuous band of spatial frequencies within a prescribed interval *k*_1_ ≤ *k*_∥_ ≤ *k*_2_.^30^ See, for instance, the real-space point distribution and structure factor of the HUDLE holes pattern (Figure 4a). The suppression of density fluctuations at large length scales produces a broad annular feature in momentum space while maintaining a “dark” central region (Figure S2).

**Figure 4:**
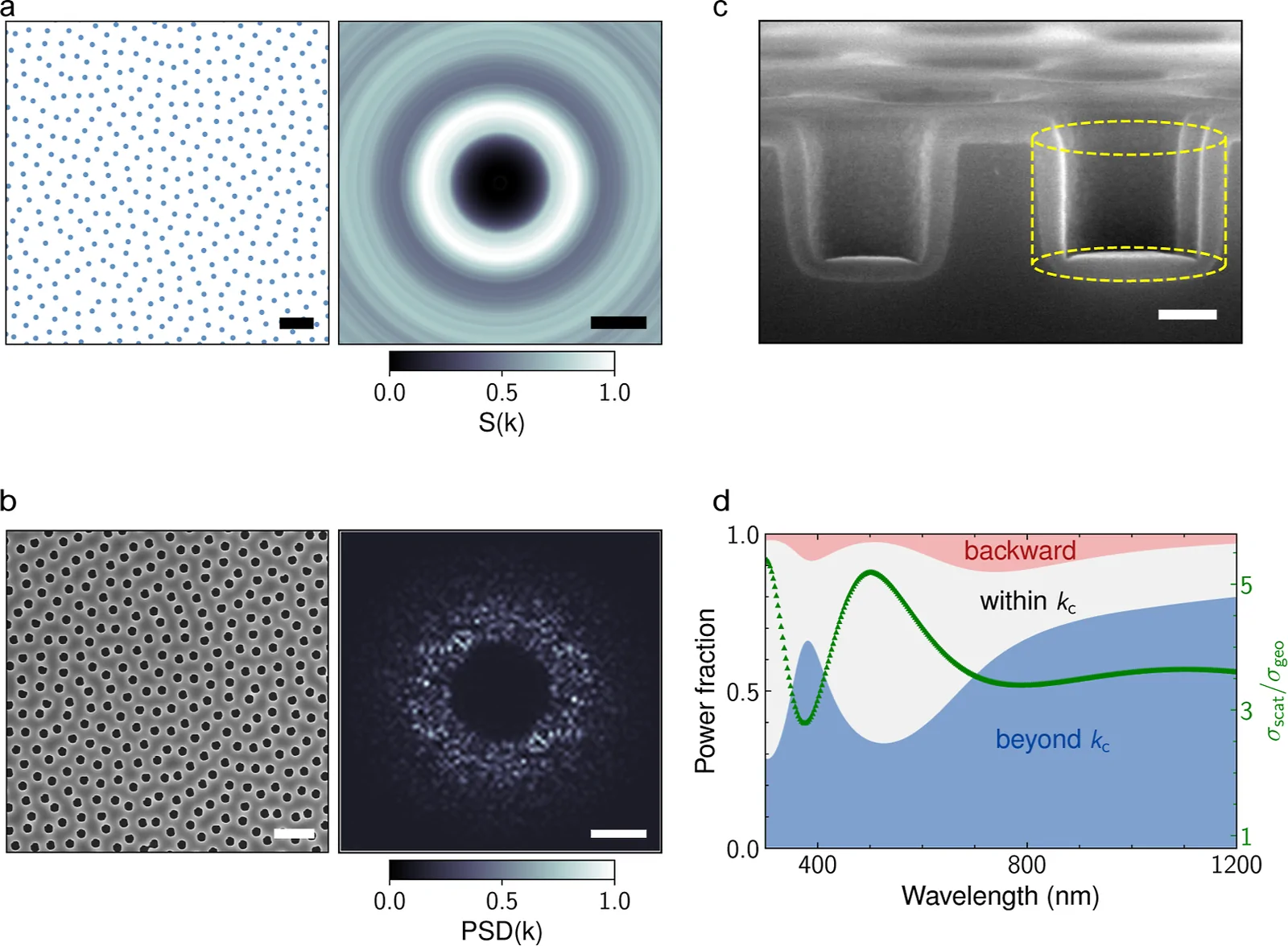
Light trapping mechanism of HUDLE layers. (a) HUDLE holes real space locations (left) and their corresponding structure factor *S*(*k*) (right). (b) SEM image of an as-fabricated HUDLE Holes pattern (left) and 2D fast-Fourier transform (FFT, right), obtained from it after Otsu thresholding and subtracting its mean intensity. Scale bars in (a, b) are 1 μm and 10 μm^–1^ for real- and Fourier space images, respectively. (c) Cross-sectional SEM image of the as-fabricated sample with the HUDLE hole pattern, where the scattering object is highlighted by a dashed frame and the scale bar is 100 nm. (d) Scattering cross-section (green triangles) and direction-resolved scattered power fractions of a typical scattering object of the HUDLE holes pattern; a hole (*n* = 1) coated with 55 nm SiO_2_ located at a silicon–air interface (*n*_Si_ = 3.5, *k*_Si_ = 0) of 180 nm radius and 245 nm height. Power fractions are displayed as shaded areas with reflection at the top (red), forward scattering into the escape cone (*k*_∥_ < *k*_c_) in the middle (gray), and forward scattering beyond the critical angle (*k*_∥_ > *k*_c_) at the bottom (blue).

When scatterers are placed at these hyperuniform locations (e.g., holes), the resulting PSD, given by the squared-Fourier transform of the real space image (Figure 4b), extends beyond the ideal HUD interval due to convolution with the scatterer form factor. Crucially, however, the suppression of small *k*_∥_ and the continuous momentum distribution are preserved, as shown by the PSD of the hole pattern in Figure 4b, right. Hyperuniformity therefore ensures both minimal small-angle scattering and broadband access to guided-mode momenta.

To elucidate the spectral dependence of this behavior, we analyze the response of the individual hole geometry embedded at the air-Si interface (Figure 4c,d). A cross-sectional SEM image of an as-fabricated HUDLE Hole sample is displayed in Figure 4c, with a dashed frame highlighting an individual hole. The calculated scattering cross section of such an individual hole, embedded in a nonabsorbing dispersionless medium (*n* = 3.5) and coated with 55 nm of SiO_2_, exhibits resonances corresponding to localized dipolar and Fabry-Pérot-like modes (green triangles in Figure 4d).

To highlight the differences in directionality, we also plot the breakdown of scattering power over the different angular ranges in Figure 4d. Importantly, a substantial fraction of the forward-scattered power is directed beyond the escape cone, particularly at longer wavelengths near the dipole resonance. This indicates that the basic scattering unit preferentially redistributes near-infrared photons into large in-plane momenta and thereby provides coupling to guided modes. However, the overall device response cannot be understood from single-scatterer behavior alone. The full optical response arises from the interplay between individual scattering resonances and the collective spatial correlations encoded in the structure factor. We therefore simulate full lateral areas of the nanopatterns at a semi-infinite air–Si interface and evaluate the *k*_∥_-resolved transmitted power (Figure 5). These simulations isolate the momentum redistribution caused by the front-surface scattering layer.

**Figure 5:**
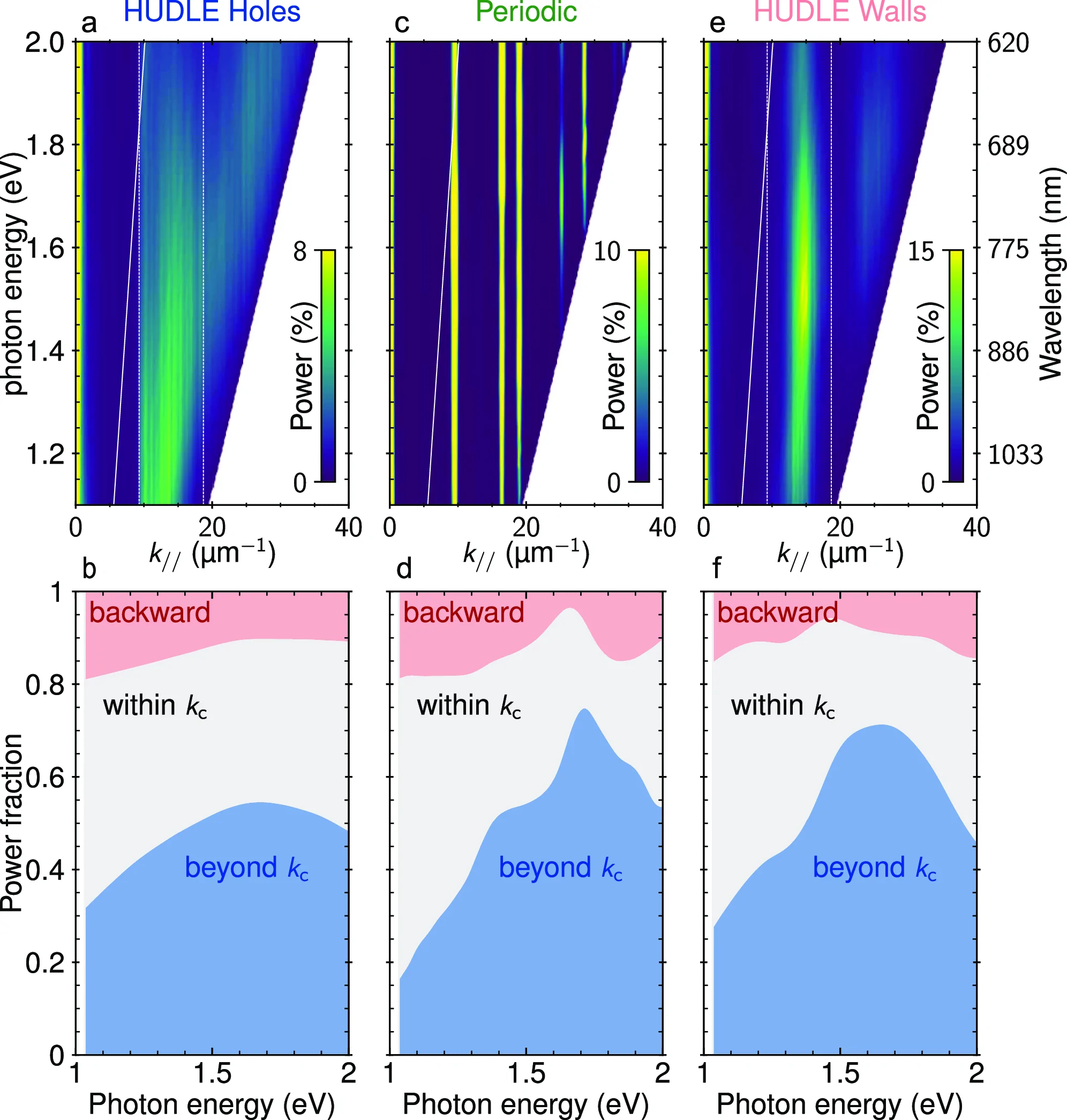
Momentum-resolved light trapping analysis of complete patterns. (a,c,e) Simulated power distribution of light transmitted through a semi-infinite air–Si interface as a function of in-plane momentum and photon energy for the HUDLE holes pattern (a, left), periodic (c, middle), and HUDLE walls (e, right). The white solid line represents the light line (critical angle) and vertical dashed lines denote in-plane momentum limits of the HUD design. (b,d,f) Integrated power fractions of light scattered by the nanostructures. Reflected power fraction (backward) shown in red at the top, power scattered into the escape cone (within *k*_c_) gray in the middle, and power scattered beyond the critical angle *k*_∥_ > *k*_c_ in blue at the bottom for HUDLE holes (b), periodic (d), and HUDLE walls (f) as a function of photon energy.

Figure 5a,c,e show the calculated fractions of scattered/transmitted power (color scale) as a function of in-plane momentum and photon energy for all nanopattern types. The HUDLE Hole pattern (Figure 5a) distributes scattered power continuously across the engineered momentum band (indicated by dotted white vertical lines) and maintains substantial scattering beyond *k*_*c*_ throughout the near-infrared range. This broad redistribution of in-plane momenta ensures sustained coupling to guided modes, even at longer wavelengths.

More specifically, the integrated power fractions (Figure 5b) show that throughout the entire spectrum, the HUDLE Hole pattern redirects more than a third of the incident power beyond the escape cone in a single pass through the scattering pattern. This fraction decreases at lower energies, which is in equal parts due to increasing reflectance and small-angle forward scattering.

In contrast, the periodic design redistributes light into a limited number of discrete diffraction orders (Figure 5c). As the wavelength increases, the number of diffraction orders beyond the light line decreases, which reduces the fraction of power coupled into guided modes and limits near-infrared light trapping. The total fraction of power scattered beyond *k*_c_ scales with the number of diffraction orders available.^18^ The consequences at small photon energies are clearly seen in the breakdown of scattered power (Figure 5d), where the power scattered beyond the escape cone drastically drops for energies <1.5 eV (830 nm).

Lastly, the HUDLE Walls (Figure 5e,f) exhibit a similar overall behavior but with the scattered power more strongly concentrated within the engineered momentum interval of the design. This corresponds to the pronounced peak in their PSD (Figure S2), more closely resembling a periodic structure. Comparing the integrated power scattered beyond the escape cone for the different designs (Figure 5b,d,f) reveals that both HUDLE patterns redistribute a larger fraction of light into *k*_∥_ > *k*_*c*_ in the near-infrared than the Periodic, consistent with their enhanced EQE. Among the two hyperuniform designs, the HUDLE Holes exhibit the highest fraction of power beyond *k*_*c*_ in the NIR, in direct agreement with their superior EQE in this spectral range.

Altogether, the combination of (i) suppressed small-angle scattering and (ii) broadband, continuous momentum redistribution overlapping with the guided-mode dispersion enables hyperuniform nanopatterns to outperform periodic designs in ultrathin absorbers. This momentum-space picture provides a direct physical explanation for the superior near-infrared EQE measured in HUDLE devices.

### Final Remarks

The device architecture presented in this work is not intended to maximize absolute power conversion efficiency. Instead, it serves as a controlled platform to directly probe front-surface scattering and guided-mode coupling in ultrathin absorbers. Despite the high material quality and excellent surface passivation demonstrated by the preserved *V*_OC_ and fill factor, the current device stack contains intrinsic limitations. The thick emitter and 2 μm seed layer introduce parasitic absorption and the lack of a reflective back interface prevents recovery of light scattered below the critical angle. The persistence of Fabry–Pérot oscillations in the long-wavelength reflectance spectra indicates that a non-negligible fraction of incident light remains in the zeroth diffraction order and is not redistributed into high in-plane momenta.

Simulations of the periodic reference suggest that replacing the buried oxide with a metallic mirror and further improving the antireflection coating would yield an additional increase in the short-circuit current of ∼7 mA/cm^2^ (resulting in a ∼3% absolute efficiency gain) (see Supporting Information, Figure S7). While these values are obtained considering the periodic structure, we expect similar improvements for the HUDLE devices.

Finally, we note that the electron beam lithography used here to create the nanopatterns is incompatible with industrially scalable solar cell fabrication. Ideally, the nanopatterns are created using vacuum-free processes at room temperature and are completed in a single step or a few steps. At the same time, large-area nanofabrication methods compatible with both our feature sizes and industrial throughput have previously been reported and implemented in solar cells.^37,38^ These include substrate-conformal nanoimprint lithography^39,40^ and engineered self-assembly methods adapted to produce the feature sizes and spatial correlations presented in this work.^41,42^

## Conclusion

In summary, we demonstrate the integration of hyperuniform disordered nanopatterns into 4.8 μm thick crystalline silicon solar cells and establish their photonic operation through combined electrical measurements and momentum-resolved simulations. By intentionally omitting a metallic back reflector, our device platform isolates front-surface scattering and enables a direct assessment of coupling into large in-plane wave vectors. We show that nanopatterning preserves the electronic collection efficiency in the devices, with comparable open-circuit voltage and fill factor.

Numerical simulations reveal that this enhancement directly correlates with an increased redistribution of optical power beyond the escape cone and broadband overlap of the hyperuniform momentum band with the guided-mode dispersion of the ultrathin absorber. Compared with periodic patterns, hyperuniform textures maintain stronger scattering into large in-plane wave vectors at long wavelengths, leading to superior near-infrared photon-to-electron conversion. Our results establish hyperuniform disorder as a robust nanophotonic strategy for ultrathin silicon photovoltaics and provide a framework for designing scattering layers tailored to specific absorber thicknesses.

## Materials and Methods

### Design Optimization

The HUDLE Walls design is generated according to the method described by Florescu et al.,^28^ and the optimization of HUDLE Holes and HUDLE Walls for ultrathin silicon solar cells is described elsewhere.^30^ The hexagonal periodic pattern with a periodicity of 765 nm is based on a ring-resonator design with dimensions tailored to support Mie resonances in the near-infrared spectral range. The pattern depth optimization was done by a rough numerical calculation of the stack absorbance as a function of depth (see the Supporting Information for further details).

### c-Si Epitaxial Growth

A 4.8 μm thick c-Si layer is grown by reduced-pressure chemical vapor deposition (RPCVD) at 1000 °C on a (100)-oriented SOI wafer. The thickness of the buried oxide layer of the SOI is 500 nm and the device (seed) layer is 2 μm. The epitaxially grown c-Si multilayer consists of p^+^/p/i/n/n^+^ doped layers of (600/300/2800/300/800) nm thickness, respectively, from the seed layer to the top of the device. Doping levels are analyzed using 4-point probe measurements and are found to be (8 × 10^19^/1 × 10^18^) cm^–3^ for (p^+^/p) with boron dopants and (5 × 10^19^/1.2 × 10^17^) cm^–3^ for n^+^/n layers with phosphorus dopants, respectively. A cross-sectional sketch of the solar cell stack is displayed in Figure S4.

### Nanostructure Fabrication

The nanostructures are fabricated using electron-beam lithography (EBL), using a RAITH Voyager system. A positive tone resist AR-P 6200 (CSAR 62, 4% in Anisole) is spin-coated on the epitaxially grown c-Si, exposed, and developed in pentyl acetate, ortho-xylene, and methyl isobutyl ketone (MIBK)/isopropanol (IPA) ratio 9:1. The reactive ion etch of Si (Lam Research TCP 9400 Poly Etcher) uses 100 sccm HBr, 40 sccm Cl_2_, 5 sccm O_2_/He at a background pressure of 6.0 mTorr, and 250 W forward RF power.

### Solar Cell Fabrication

Individual cells are galvanically isolated by a sequence of optical lithography using a Heidelberg instrument MLA 150, followed by resist development and Si etch of 4.3 μm to reveal the p^+^-doped layer at the base of the solar cell stack. We use the same etch recipe as above. After mesa etching and resist removal, the wafer is placed into a dry oxidation oven at 1000 °C to grow 55 nm silicon dioxide on all exposed Si surfaces. Next, 80% of the wafer is covered while the exposed regions receive 31 nm of SiN_*x*_ using plasma-enhanced chemical vapor deposition (PlasmaTherm Shuttlelock). This thickness is chosen to minimize reflection while accounting for the 55 nm SiO_2_ underneath. Front contact regions are defined using optical lithography (Heidelberg system). Subsequently, the SiO_2_ (and SiN_*x*_) in the contact regions is removed using 20:1 buffered oxide etch. Electron-beam evaporation is used to deposit the contact layers consisting of Ti/Al (10/300 nm). Subsequently, the same procedure is repeated to deposit NiOx/Pt/Ag (5/40/160 nm) as the back contact layers. Finally, the wafer is placed in a rapid thermal annealer (RTA AllWin 610) in forming gas at 450 °C in order to reduce contact resistance between the Si and metal layers. The cells’ active areas measure (0.0196 ± 0.0004) cm^2^ without front metal contacts, obtained through calibrated microscopy images (see Figures S8–S12). Only the active area is used for calibration of the current density and justified by additional EQE measurements with light excitation only on the bus bars, contacts, and next to the cell mesa (Figure S6), which show negligible current contribution from metalized or galvanically isolated regions.

### *J*–*V* Curve Measurements

We use a Newport Solar simulator (94062A Class ABA) as a source that outputs an AM1.5G spectrum. The intensity is close to one sun but can vary by a few percent. The intensity is measured using a calibrated solar cell (Newport Oriel 91150 V Reference Cell and Meter) before and after each measurement of every device. Current measuring and voltage sourcing are performed by a source-measure unit (Agilent B2902A). Measurements are performed with two leads which combine voltage sourcing and current sensing through the same contact point. Each cell’s *JV*-curve is measured four times in direct succession. Selected cells are also measured in a different setup using a G2 V solar simulator and Keithley 2400 SMU and show power conversion efficiencies within the range of uncertainty of the first setup.

### Dark-*JV* Measurements

We use a Keithley 2450 SMU and four point probes to perform the *JV* measurements in the dark. The voltage is swept manually with an array of set voltage points since the currents are close to the sensitivity limit of the device of ∼10 fA. At each point, the voltage drop across the cell is measured and then multiple current measurements with 200 ms integration time are performed. Measured values that are within 2% of each other are averaged and others discarded. If the current measurement deviates by more than an order of magnitude from the adjacent current points, it is likewise discarded. The obtained spectra are analyzed using the 2-diode-model^43^ to extract the dark saturation current densities as well series and shunt resistance.

### External Quantum Efficiency

The EQE setup is custom-built using a Witec microscope, a supercontinuum laser (NKT), and an acousto-optic tunable filter (NKT SuperK Select). A preamplifier (Stanford Research Systems SR570) and a lock-in amplifier (Stanford Research Systems SR830) are used for current measurements. The incoming laser is periodically interrupted by a chopper wheel rotating at (40–65) Hz. The spot size measures approximately 50 μm in diameter and is placed in the center between the metal fingers. No optical or electronic biasing is applied. The optical power at the sample position is measured with a power meter (Thorlabs PM100D), and the electronic readout circuit is gauged with a calibrated Si photodiode (Thorlabs FDS1010-CAL). The AM1.5G spectrally averaged and integrated EQE spectra fall short of the measured *J*_SC_ from *JV* measurements but retain the relative difference between patterning types. This is partly due to the inaccessibility of the full wavelength range necessary for faithful comparison and partly to the absence of biasing. The latter reduces effectiveness of charge-separation by the p–i–n junction as well as conductance in the highly p-doped layer at the cell base, which also transports carriers laterally to the metal back contacts located at the circumference of the cell. As a consequence, there is increased recombination in the dark regions of the cells. Since this systematic error is equivalent for all pattern types and measurements, the relative EQE comparison remains valid.

### Structure Factor

The (static) structure factor in this work is defined^26^

1 S(k⃗)=1+ρh̃(k⃗)

where ρ denotes the number density in the sampling window and h̃ is the Fourier transform of the total correlation function

2 $$h\left(r\right)=g_{2}\left(r\right)-1$$

with pair correlation function

3 $$g_{2}\left(r\right)=\frac{1}{\rho }\left\langle \sum_{i\neq 0}^{N}\delta \left({r-r}_{i}\right)\right\rangle$$

with ρ again denoting the number density, number of particles *N*, ensemble averaging ⟨·⟩, and the distance (**r–r**_*i*_) between particles 0 and *i*. Note that in this definition of *S*(*k*), the forward scattering associated with *k* = 0 is omitted.^44^

### Absorptance Simulations

Absorptance simulations shown in Figure 3 are performed using the rigorous coupled-wave analysis (RCWA) method using the RCWA implementation in ANSYS Lumerical.^45^ The simulation volume includes a 6 × 6 μm^2^ excerpt of the HUDLE patterns designs. The accuracy in RCWA simulations depends on the chosen number of wavevectors *N*_k_. We choose *N*_k_ > 3500 as a compromise between simulation time and accuracy. The error in *J*_sim_ compared with *N*_k_ = 3900 measures ∼0.05 mA cm^–2^ (see Figure S7).

### Angular-Resolved Scattering Analysis

The simulations shown in Figures 4 and 5 are performed using the finite-difference time-domain (FDTD) simulation software (ANSYS Lumerical 2023 R2.3). The scattering cross-section of a single hole (Figure 4d) is obtained using a total-field/scattered field (TFSF) source with large lateral dimensions 4 × 4 μm^2^ and collected using a 3D monitor box from which all scattered intensities are integrated. For the power fractions in Figure 4d, only the monitors parallel to the source-plane are used, which capture most of the forward/backward directed scattering power independently. Through far field transformation of the near fields captured by the monitor inside the substrate, the in-plane momenta of the scattered intensities are obtained and integrated for the ranges within and without the escape cone.

For the full HUDLE pattern simulations (Figure 5), the transmitted powers are also obtained through FDTD, where we simulate a single pass through the scattering layer. The simulation boundaries are perfectly matched layers (PML) in the directions normal to the air-Si interface. In the lateral direction, we utilize approximately 15 × 15 μm^2^ area to include the entire nanopattern design. The large lateral dimension is necessary due to periodic boundary conditions applied here, which for smaller areas give rise to unrealistic diffraction orders. The simulation mesh is generated using auto nonuniform conformal meshing with cubic Yee cells of side lengths ≥ (4 nm)^3^. In the case of the periodic pattern, a single unit cell is simulated and for the far field analysis, a number of unit cells is chosen such that the total lateral area is approximately equal to 15 × 15 μm^2^, which facilitates faithful comparison with the hyperuniform designs. Simulations are performed with a spectral resolution of 3 nm and convergence is assumed once the total power residing in the simulation volume has decreased to 10^–5^ of its initial value either through absorption in the material or by the PML.

## Supplementary Material



## Notes

Absorption limits; Power Spectral Density plots; nanopattern depth optimisation; solar cell stack schematics; IV curves analysis; comparing simulated absorptance, EQE and IV curves; absorptance simulations of the real and optimized device stacks; and solar cell pictures (PDF)
