# Supplementary material for: Integrating Hyperuniform Disordered Light Trapping Layers in Ultrathin c‑Si Solar Cells
Source: ACS Photonics. 2026 Oct 12;13(20):6104–13. doi: 10.1021/acsphotonics.6c01224 (PMC13693493; doi:10.1021/acsphotonics.6c01224)
Supplement: Supplementary file 1 [file ph6c01224_si_001.pdf]

Supplementary Information: Integrating hyperuniform disordered light trapping layers in ultrathin c-Si solar cells

Alexander Lambertz[1,2], Nayeun Lee[3], Ali P. Vafa[4], Viktoryia Shautsova[3], Fenghao Xu[3], Jung-Hwan Song[3], Nicholas A. Güsken[3], Skyler Selvin[3], Marian Florescu[4], Mark Brongersma[3], Jorik van de Groep[2*], Esther Alarcon-Llado[1,5*]

[1]Center for Nanophotonics, AMOLF, Science Park 104, 1098XG, Amsterdam, The Netherlands.
[2]Van der Waals–Zeeman Institute, Institute of Physics, University of Amsterdam, Science Park 904, 1098XH, Amsterdam, the Netherlands.
[3]Department of Materials Science and Engineering, Stanford University, 496 Lomita Mall, Stanford, CA 94305, USA.
[4]Optoelectronics Research Centre, University of Southampton, Southampton SO17 1BJ, UK.
[5]Van 't Hoff Institute for Molecular Sciences, University of Amsterdam, Science Park 904, 1098XH, Amsterdam, the Netherlands.

*Corresponding author(s). E-mail(s): j.vandegroep@uva.nl; e.alarconllado@amolf.nl;

Contents

1 Absorption limits 3

2 PSD Profiles 4

3 Nanopattern depth optimisation 5

4 Solar cell stack schematic 7

5 Double-Diode Model 8

6 Comparing simulated absorptance, EQE and IV curves 9

7 Absorptance simulations of the optimized design 10

8 Solar cell micrographs 11

1 Absorption limits

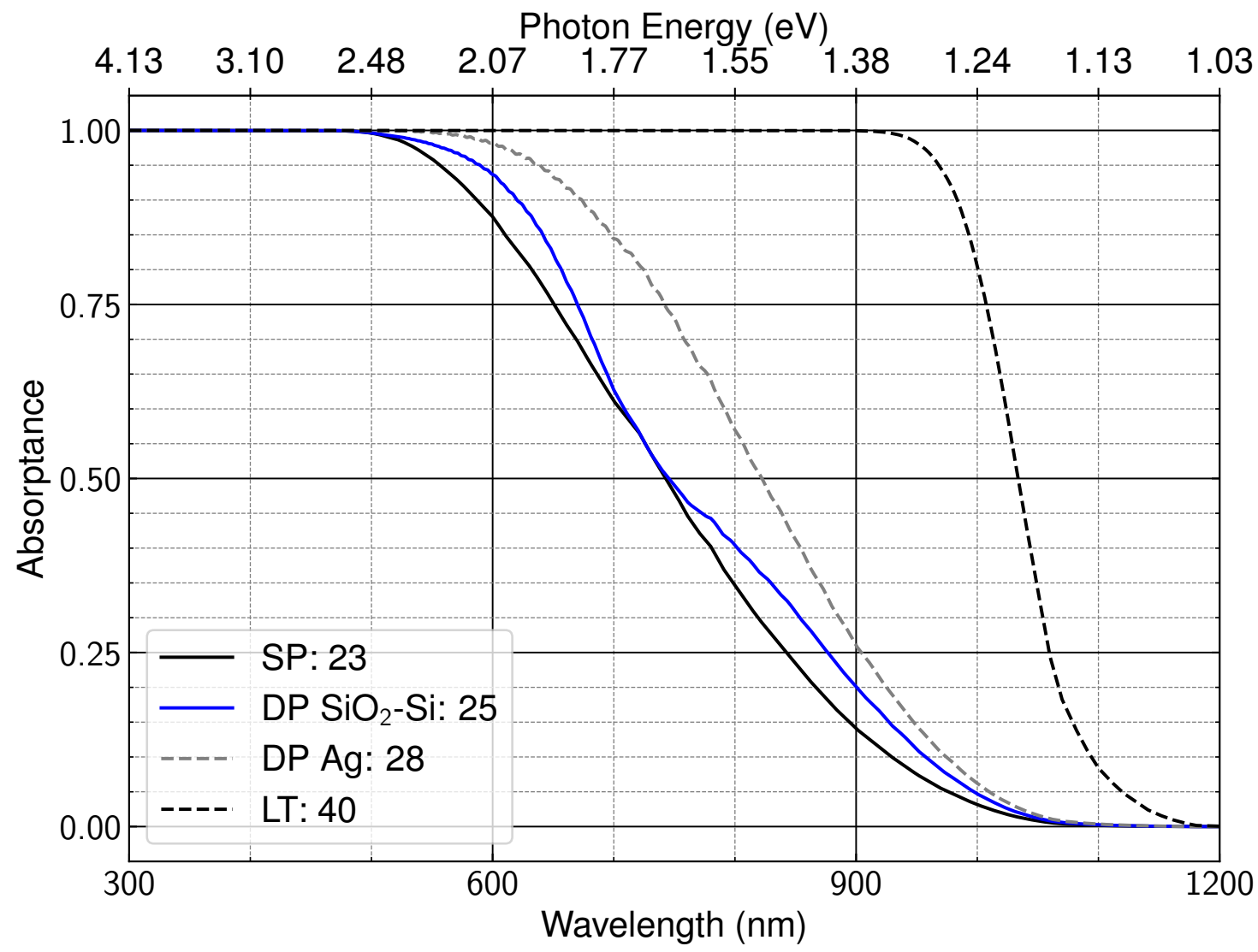

Fig. S1 Absorption limits in 5 µm silicon slab.

The fraction of absorbed light as a function of wavelength for a single pass (SP), double pass with SiO_2/Si reflector in SOI (DP SiO2-Si), double pass with Ag reflector (DP Ag), and lambertian light trapping (Yablonovitch $4n^2$ limit [1], LT). Legend shows AM1.5G-averaged J_{SC} in mA/cm^2.

Figure S1 shows the absorptance spectrum in 5 µm Si assuming no front-surface reflectance and either a silver back reflector at the rear (Ag) or the reflectance of the SiO_2/Si interfaces of the SOI wafer (SiO_2-Si), calculated using transfer matrix method (TMM) under normal incidence of coherent light. At around 720 nm, the double-pass within the SOI structure coincides with the single-pass absorption limit due to a standing wave (Fabry-Pérot resonance) inside the buried oxide layer of the SOI, which suppresses reflection back into the absorbing layer. This nodal point is likewise observed in the EQE and reflectance measurements (Fig. 3 in the main text).

2 PSD Profiles

Figure S2 displays real- and momentum-space images of the pattern designs. The 2D FFTs (middle row) are calculated from the shown $15 \times 15\,\mu m^2$ excerpts of the images in the top row. As a consequence, the resolution of the FFT and its radial average (bottom row) limited by the relatively lateral dimensions.

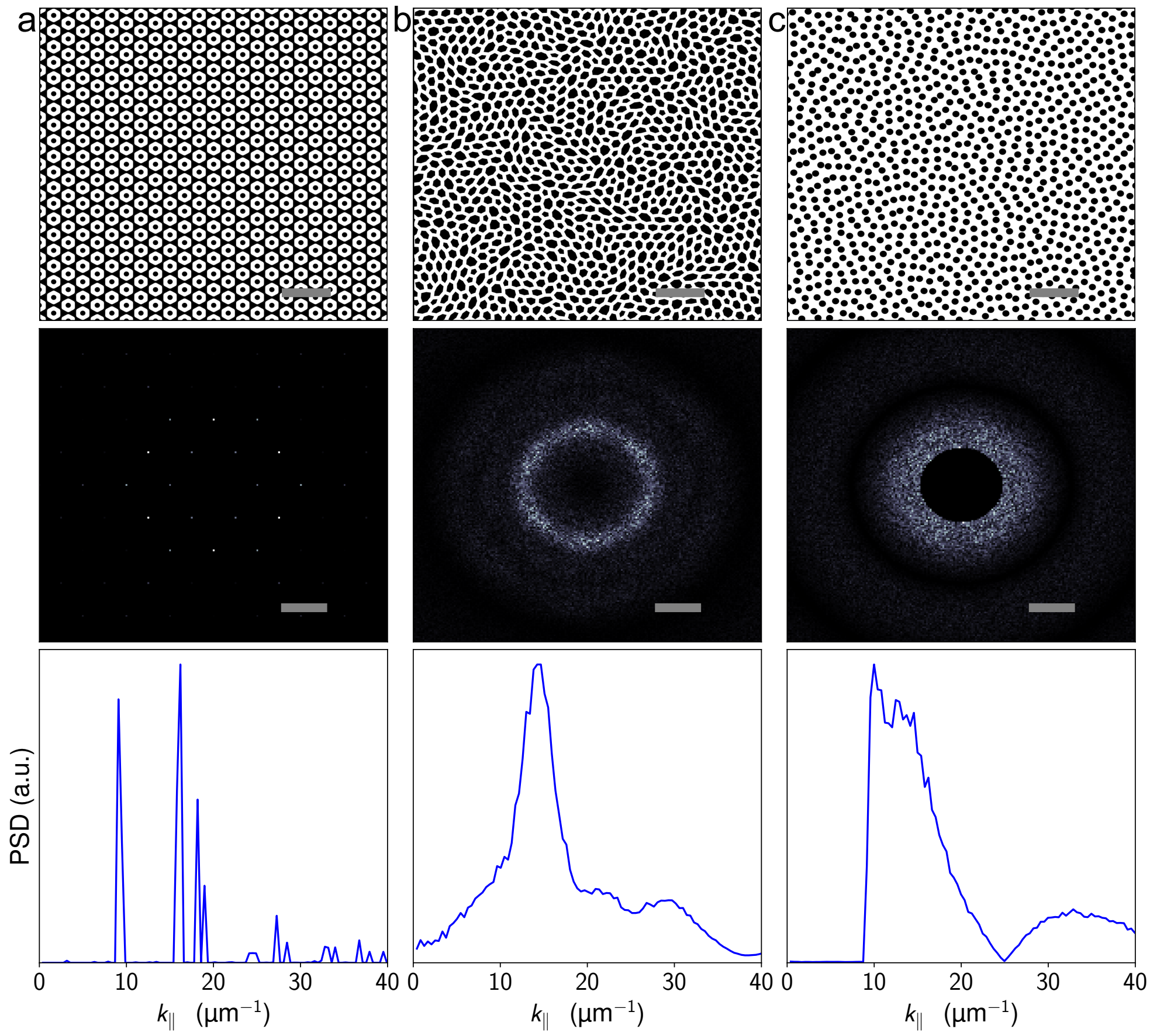

Fig. S2 Power spectral density of nanopatterns
*Real-space image (top row), 2D FFT (middle row) and radial average of FFT (bottom row) of Periodic (**a**), HUDLE Walls (**b**), and HUDLE Holes (**c**). Scale bars are 2 µm and 10 µm^{-1} top and bottom row, respectively. White regions in the top row are protruding.*

3 Nanopattern depth optimisation

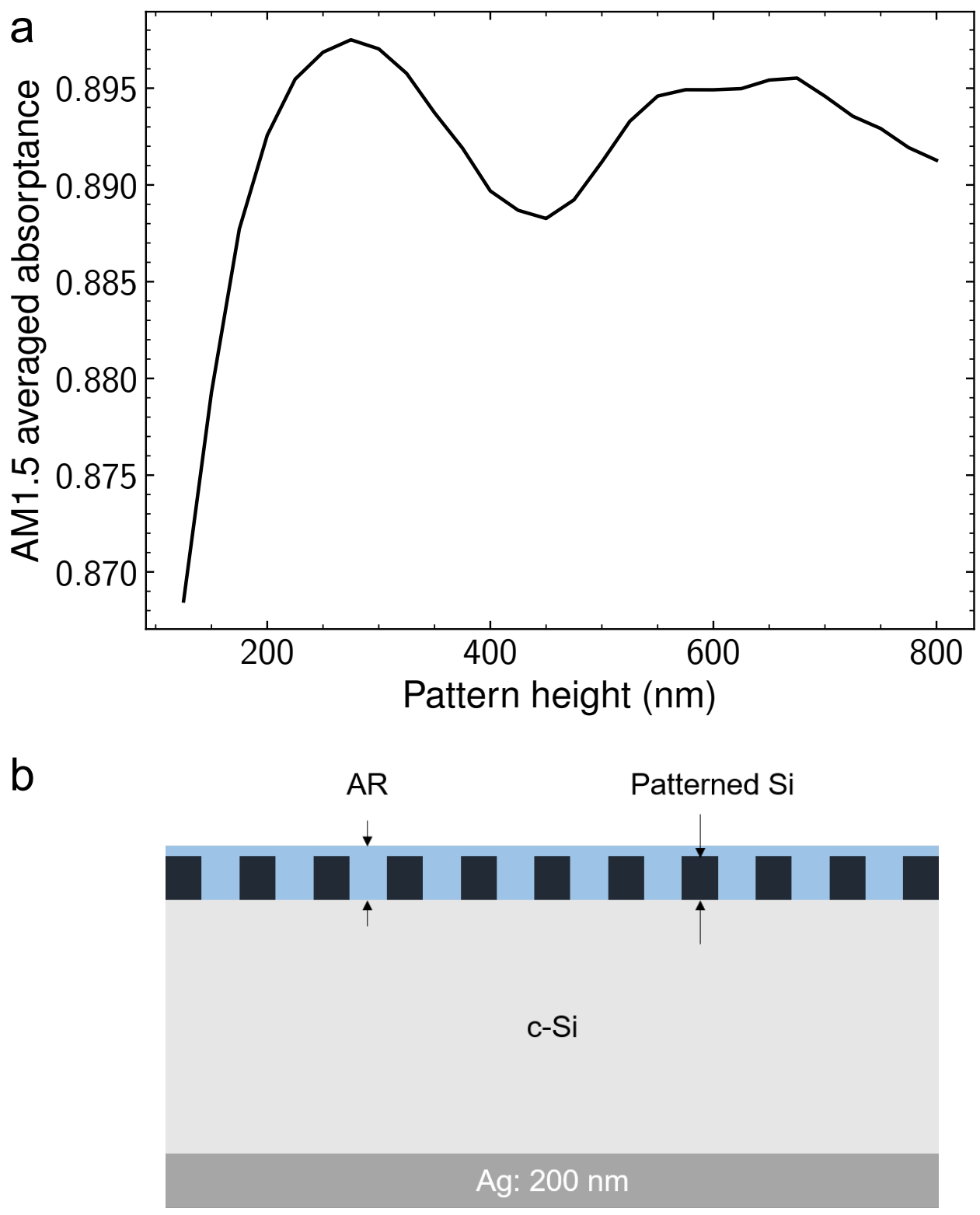

Fig. S3 FDTD-simulated absorptance versus nanopattern height *h*.
(**a**) AM1.5G-averaged absorptance ($\lambda \in$[300,1200] nm) of a 5 µm-thick Si slab on 200 nm Ag for varying heights h of a HUDLE layer etched into it's top surface. (**b**) Schematic of the simulation volume. The bulk silicon thickness $d_{\mathrm{Si}} = 5\,\mu\mathrm{m} - h$. The void regions within the nanopattern are infiltrated with an anti-reflective coating (n=1.81) of thickness d_{AR}=h+72 nm that extends 72 nm above the nanopattern.

Figure S3 shows the numerically calculated AM1.5G-averaged absorptance as a function of nanopattern depth for a representative disordered structure. The calculation was performed for a 5 μm-thick Si slab with an Ag back reflector and an idealized anti-reflection coating, and was used to identify an appropriate fabrication range rather

than to optimize the final HUDLE Holes and HUDLE Walls device geometries individually. The absorptance was calculated by using FDTD as described in the main text methods, with the exception that in this sweep, a coarse mesh of $(20\,\mathrm{nm})^3$ is used in the homogeneous regions and $(8\,\mathrm{nm})^3$ Yee cells in the patterned regions.

The absorptance exhibits a broad maximum for pattern depths of approximately 250–300 nm. Based on this result, a nominal depth of 255 nm was selected as the fabrication target. The resulting fabricated structures had a depth of approximately 245 nm, which remains within this broad optimum and differs slightly from the target value due to fabrication tolerances. The representative structure used for this depth sweep corresponds to a spinodal design included on the same fabrication wafer. Although this design was subsequently excluded from the device comparison because of fabrication failure during the etching step, it provides a suitable representative geometry for establishing the relevant nanopattern-depth range.

4 Solar cell stack schematic

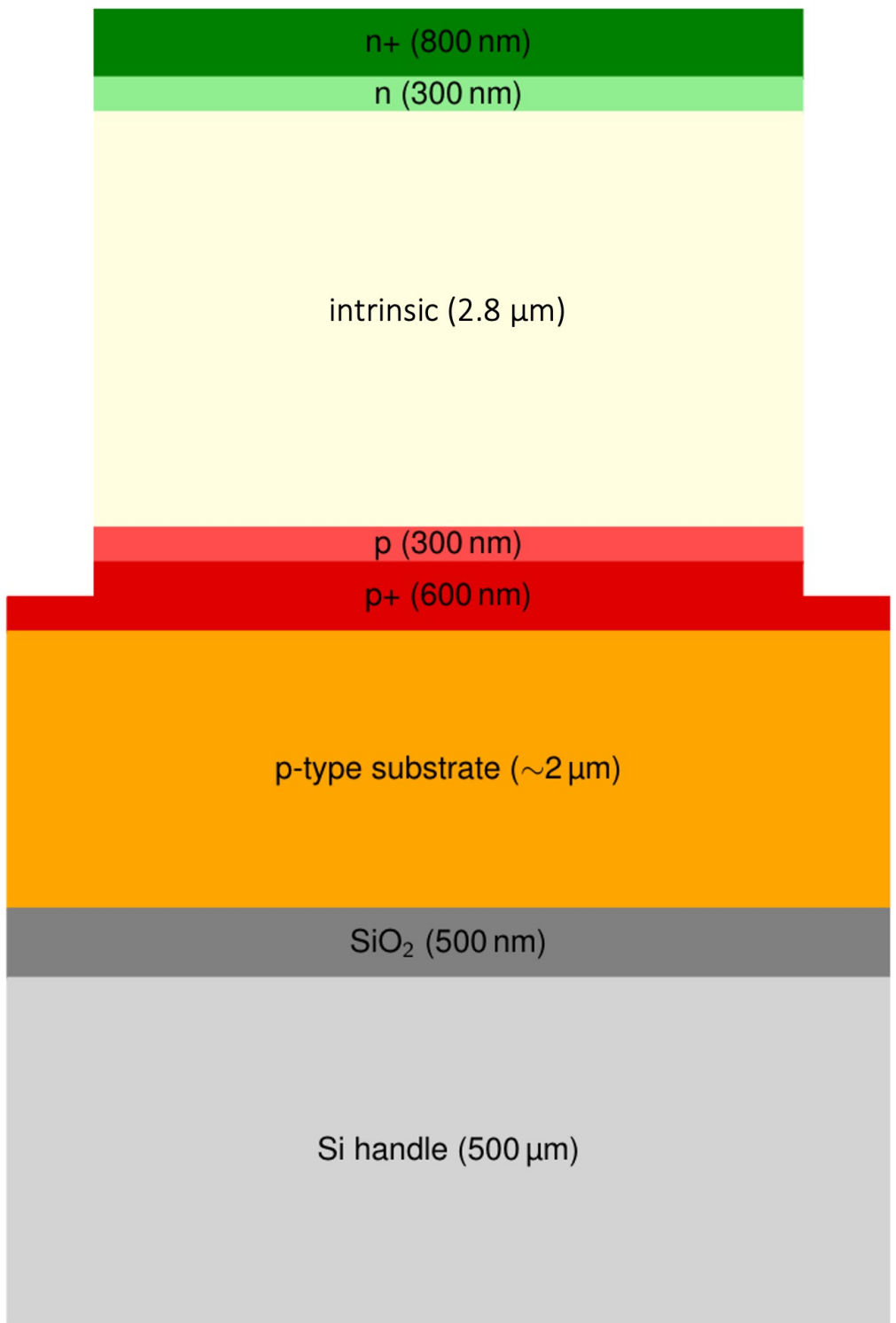

Fig. S4 Solar cell stack on SOI.

Schematic of the as-grown solar cell stack on SOI platform. The slimmer top layers represent the mesa region, which is defined by optical lithography and etched to reveal the p+-region at the base. The latter also serves as the lateral carrier transport layer to the metal back contacts which are evaporated into the etched trenches.

5 Double-Diode Model

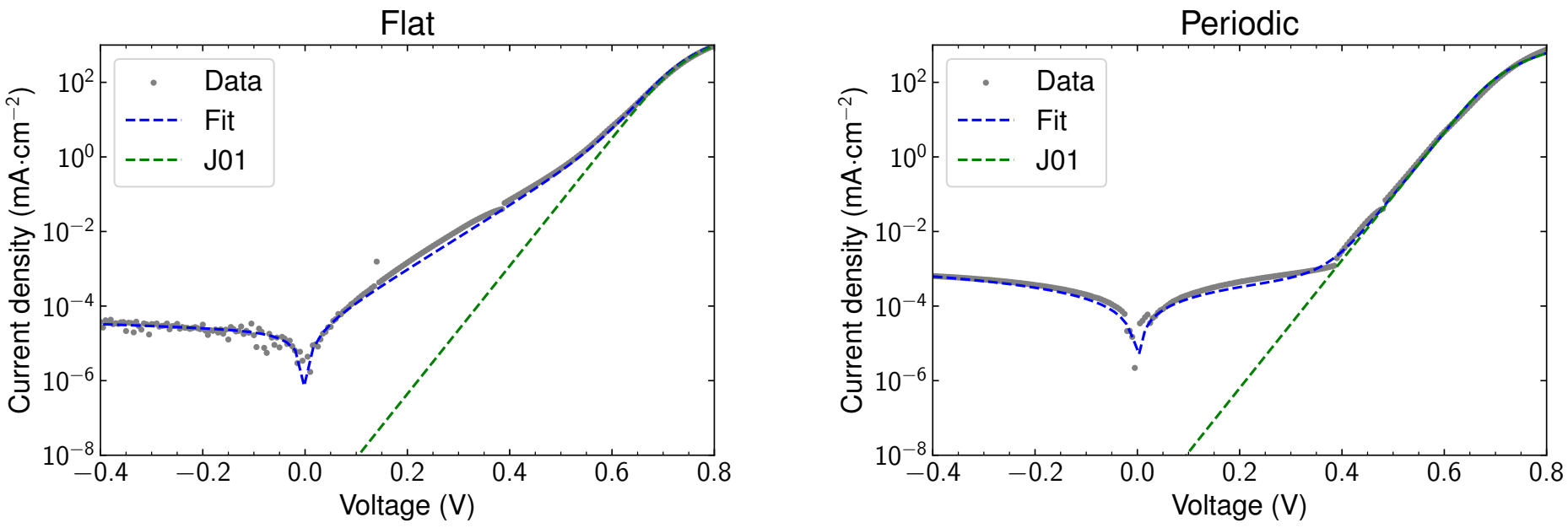

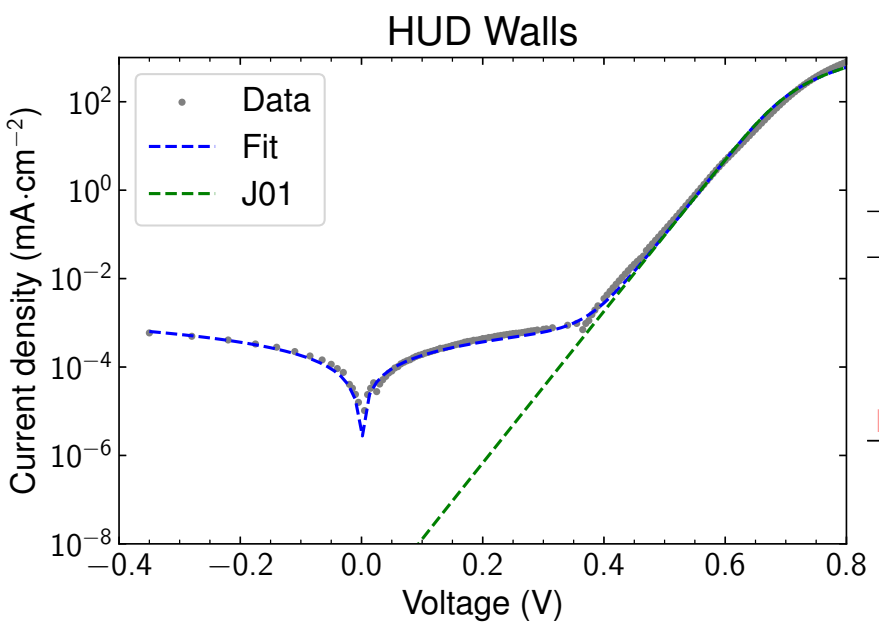

Table S1: Extracted parameters from 2-diode model

Pattern	J_{01} (fA cm^{-2})	J_{02} (mA cm^{-2})	$R_{parallel}$ (Ω cm^2)	R_{series} (Ω cm^2)
[18] Double	216	7.7 x 10^{-7}	1.36E+08	0.14
Flat	183 ± 28	(1.4 ± 0.7) x 10^{-5}	(2.8 ± 0.7) x 10^{7}	0.06 ± 0.01
Periodic	161 ± 26	(1.0 ± 1) x 10^{-7}	(3.9 ± 0.9) x 10^{5}	0.09 ± 0.01
HUDLE Walls	243 ± 62	(9.8 ± 1) x 10^{-8}	(5.6 ± 0.1) x 10^{5}	0.13 ± 0.01

Fig. S5 2-Diode model fitted to dark J-V measurements of flat, periodic and HUD devices and a table S1 of parameters extracted from the model. The plots show the measured data as grey dots, the 2-diode-model fit as blue dashed, and the extracted J_{01} diode with ideality factor $n \equiv 1$ as dashed green curves. The uncertainty is taken from the standard deviation of fit parameters from multiple measurements on the same cell type weighted by (1-RMSE), with the root mean square error RMSE obtained from each fit. The first row in S1 [18] Double is taken from earlier measurement on the same platform but with only 3 µm absorber thickness, which was patterned with a multi-resonant nanoblock array [2].

Figure S5 shows measurements of J-V curves without illumination and a 2-diode model fit. The fit parameters are listed in table S1. [18]Double corresponds to a 3 µm-thick solar cell fabricated using the same equipment and processes and features a multi-resonant nanoblock array [2] (main text reference [18]). The fit parameter J_{01} corresponds to recombination processes in the bulk and at the surface of the device.

Since the values are very similar among flat and patterned devices of this work as well as the 3 µm device [18]Double, it shows that the patterning of the surface followed by thermal oxidation does not notably increase the recombination velocity at this interface. The ideality factor for diode J_{02} is set to 2. Due to noise in the measurement apparatus, the fit of J_{02} is suboptimal and varies substantially among the shown devices.

6 Comparing simulated absorptance, EQE and IV curves

We note that the short-circuit current predicted by integrating the simulated device-stack absorptance (shown in the main text) is several mA cm^{-2} lower than the values obtained from unmasked J–V characterization. Some of this discrepancy may arise from uncertainty in the optical constants and oxide thicknesses, but we primarily attribute it to the active-area definition and illumination geometry. The current density is normalized to the exposed mesa area, excluding regions covered by the metal grid (see also SI section 8). Local EQE measurements with a focused spot show negligible photocurrent when directly illuminating the metalized or galvanically isolated regions (see Fig. S6), supporting this area definition under the EQE measurement conditions.

Based on the local EQE measurements, the direct contribution of the covered areas to J_{SC} is on the order of 0.2%, which is less than the found discrepancy However, the illumination used in the J–V characterization may contain a diffuse component. In that case, mesa sidewalls and regions below or near the metal contacts could contribute additional photocurrent, effectively increasing the measured current density. Because the same device geometry and area convention are used for all pattern types, the relative trends remain meaningful, but the absolute J_{SC} and efficiency values should be interpreted with this systematic uncertainty in mind.

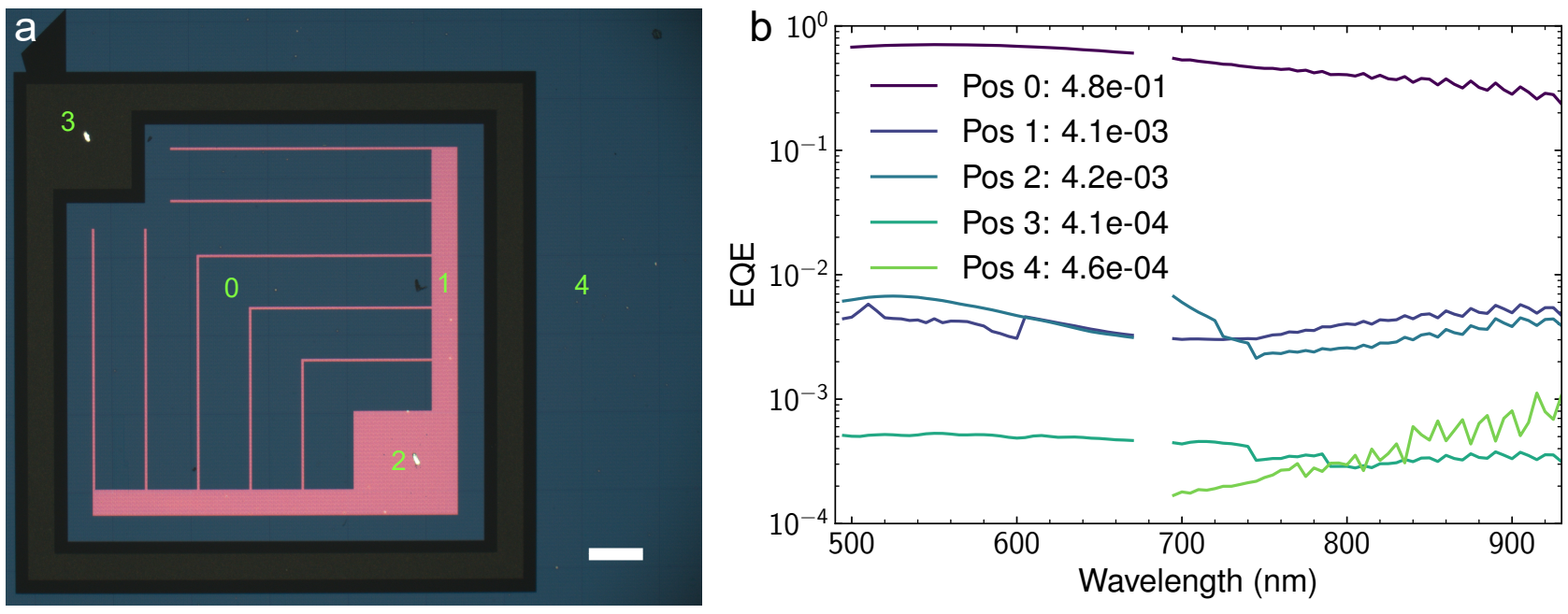

Fig. S6 EQE at different positions
a *Calibrated microscope image showing the different spot locations. Scale bar 200 µm.* ***b*** *External quantum efficiency versus wavelength at the different positions shown in (a). Visible range at position 4 produced no stable lock-in signal. Contributions from the backside contact and the galvanically-isolated surrounding regions are therefore negligible. Device type: HUDLE Holes.*

7 Absorptance simulations of the optimized design

Figure S7a displays the simulated absorptance spectra of our device stack with the periodic pattern and an optimized design. The optimized design consists of 4.8 µm-thick c-Si with the periodic pattern etched into the top 245 nm. The patterned region is infiltrated with an anti-reflective coating (n=1.81), which extends 72 nm above the top Si surface, and an Ag mirror (200 nm thick) is located directly underneath the c-Si layer (see schematic in b). The AM1.5G-averaged integrated spectra amount to 25.5 mA/cm^2 and 32.5 mA/cm^2 (range 300−1200) nm for the fabricated and optimized devices, respectively. The mirror and improved AR thus result in a gain of 7 mA/cm^2. For comparison, we also include the Double-pass and Lambertian limit spectra. Their integrated absorption leads to 28 mA/cm^2 and 40 mA/cm^2, respectively. The convergence test in (b) shows a difference of about 0.047 mA/cm^2 when the number of k-points num_k in the RCWA is increased from num_k ∼3500 to num_k ∼3900. Since this error is less than 0.2 %, simulations are performed with $num_k \sim 3500$.

Assuming otherwise unchanged values of V_{oc} and fill factor (main text Fig. 2a), an additional ∼ 3 % absolute efficiency gain would be expected.

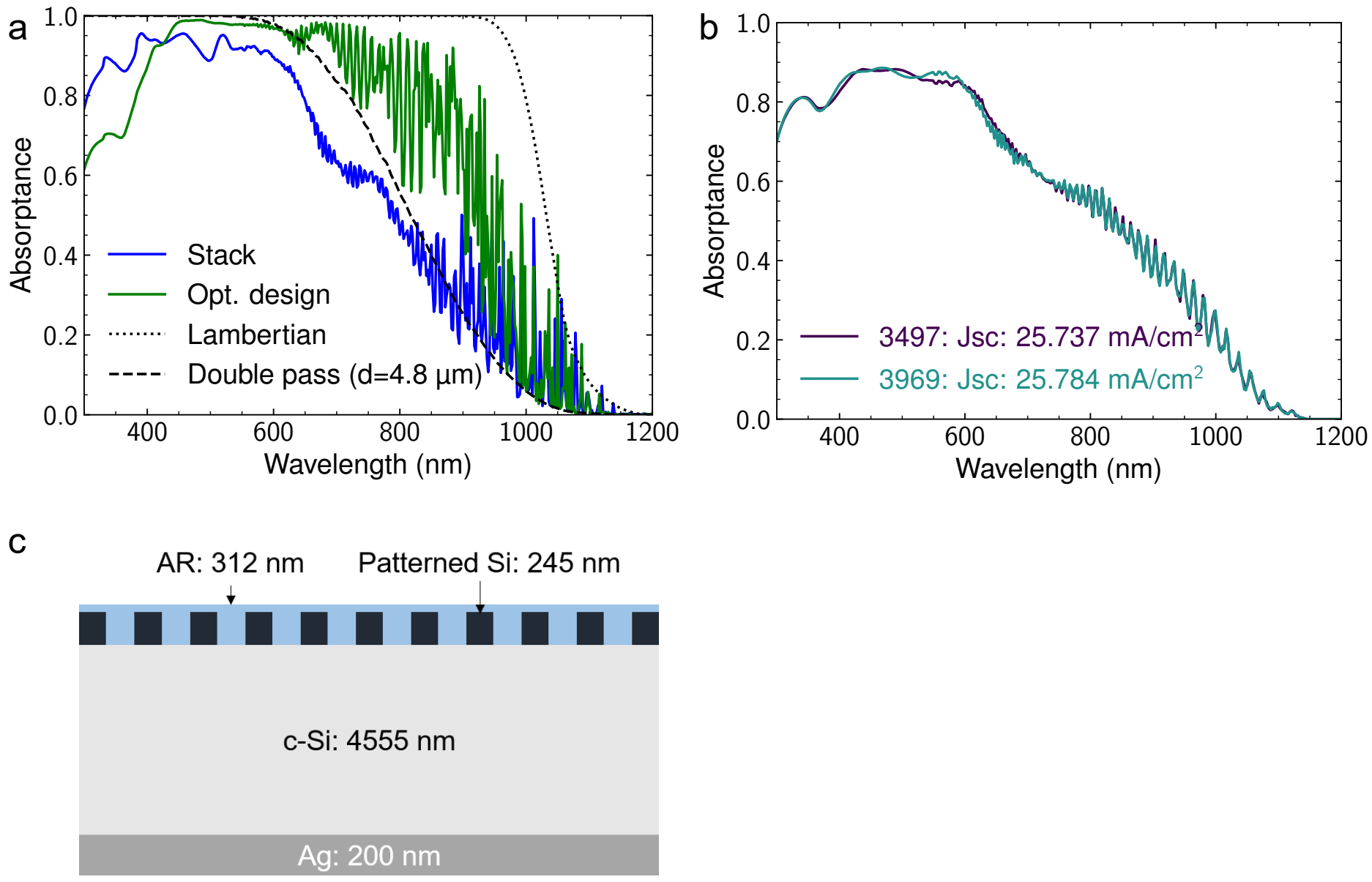

Fig. S7 Simulated absorptance of the periodic nanostructure for the SOI stack and optimized design.
*(**a**) Absorption spectrum of the simulated device stack and an optimized design with 4.8 µm Si on an Ag mirror. (**b**) Convergence testing of the RCWA chosen number of k-points num_k given in the legend. (**c**) Schematic of the optimized design, which replaces the front SiO_2 with a lossless, dispersion-free anti-reflective (AR) coating with height 312 nm, omits the parasitic seed layer, and terminates the active c-Si layer of 4.8 µm with 200 nm of Ag.*

8 Solar cell micrographs

Calibrated bright-field microscope images of each of the five cell types: Flat, SiN coated, Periodic, HUDLE Holes, and HUDLE Walls are shown.

Images are taken after characterization and thus show scratches in the contact pads from the probe contacts and possibly dirt or damage that may or may not have been present during the measurements. In the case of a nanopatterned or SiN-coated device, the entire cell region and the surrounding surface is nanostructured or coated, including the back contact metal regions and beyond. This is visible by the different background colors of the different cell types. Outer cell dimension including back contact region measures $2 \times 2\,\text{mm}^2$. The inner cell dimensions measure $1.6 \times 1.6\,\text{mm}^2$ and subtracting the top left corner of $0.3 \times 0.3\,\text{mm}^2$, the total cell area becomes $2.47\,\text{mm}^2$. The metalization from bus bars, fingers and the front contact patch in sum

covers $(0.51 \pm 0.04)\,\mathrm{mm}^2$, leaving an active area of $(1.96 \pm 0.04)\,\mathrm{mm}^2$. The error for the metal coverage results from measuring all cells using an algorithm of masking and thresholding as well as manual masking of the metal areas and cell device.

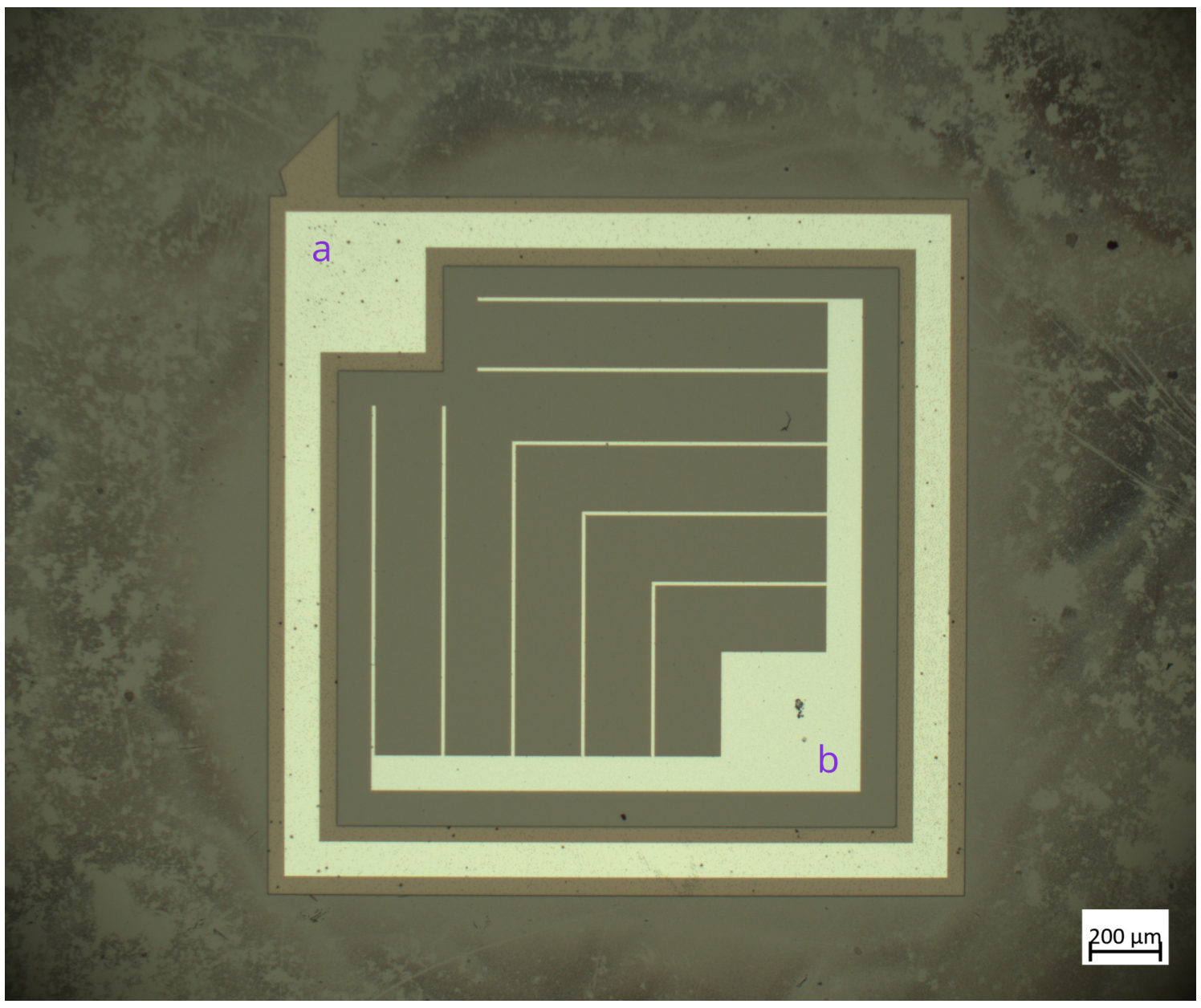

Fig. S8 Flat device. The back contact metalized region is located in a trench circumscribing the cell mesa with a large p-contact patch at (**a**). The front metal grids consist of fingers joining on the face of the cell and connected to larger bus bars at two sides of the mesa and at their junction a larger n-contact patch is placed (**b**).

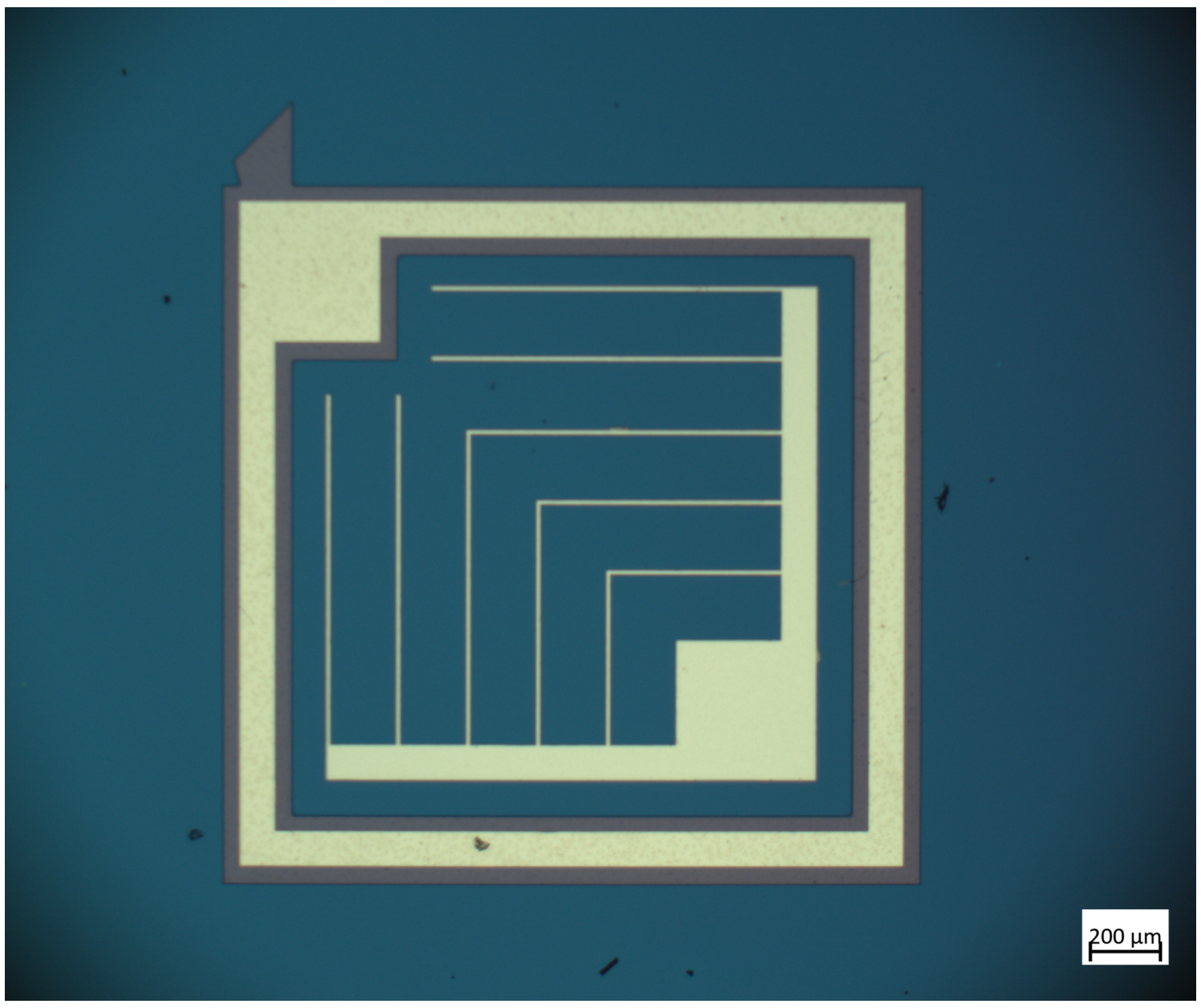

Fig. S9 Silicon-nitride-covered flat device.

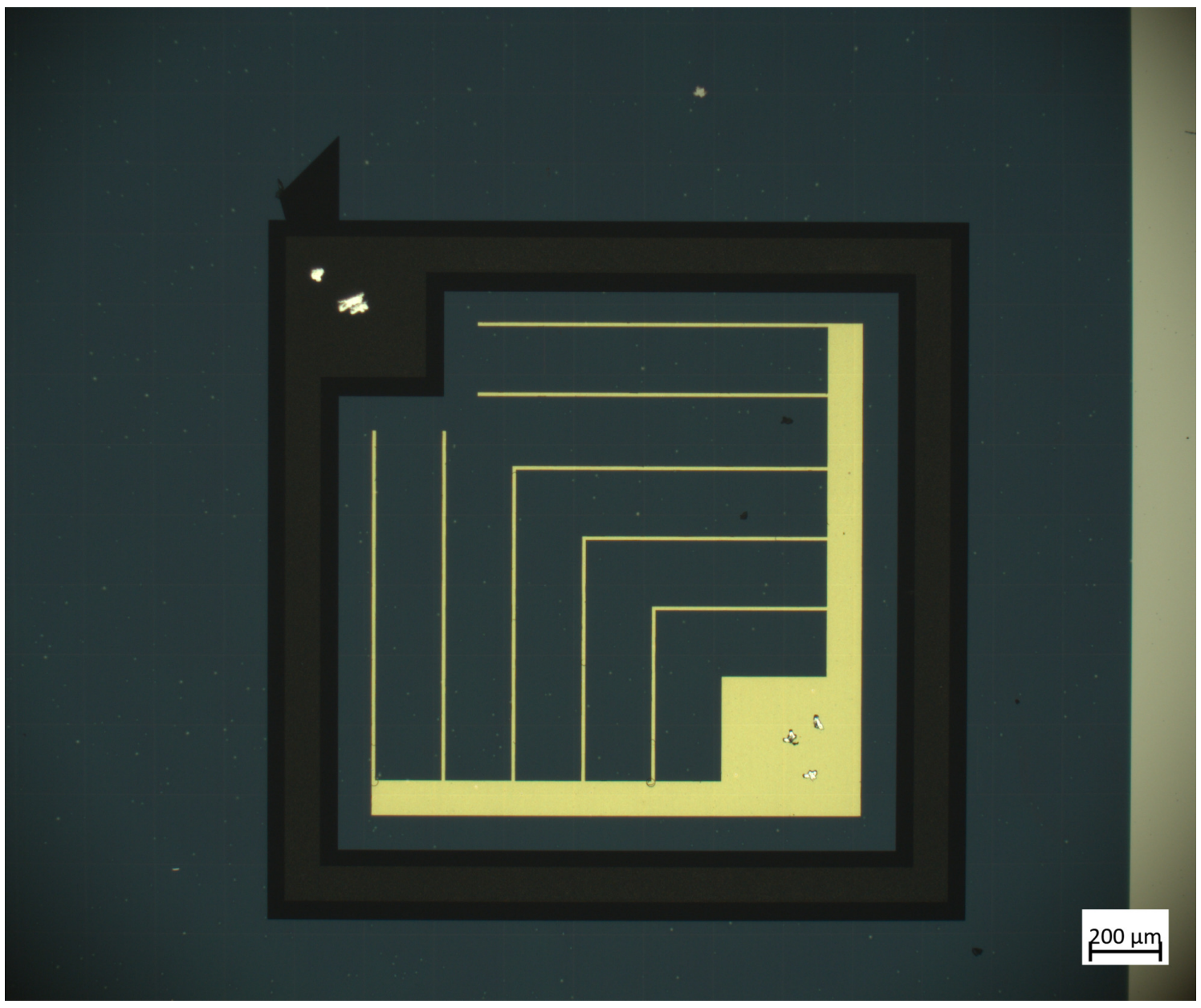

Fig. S10 Device with periodic nanostructures. The metal contacts evaporated onto the patterned cell exhibit a structured color appearance in reflection.

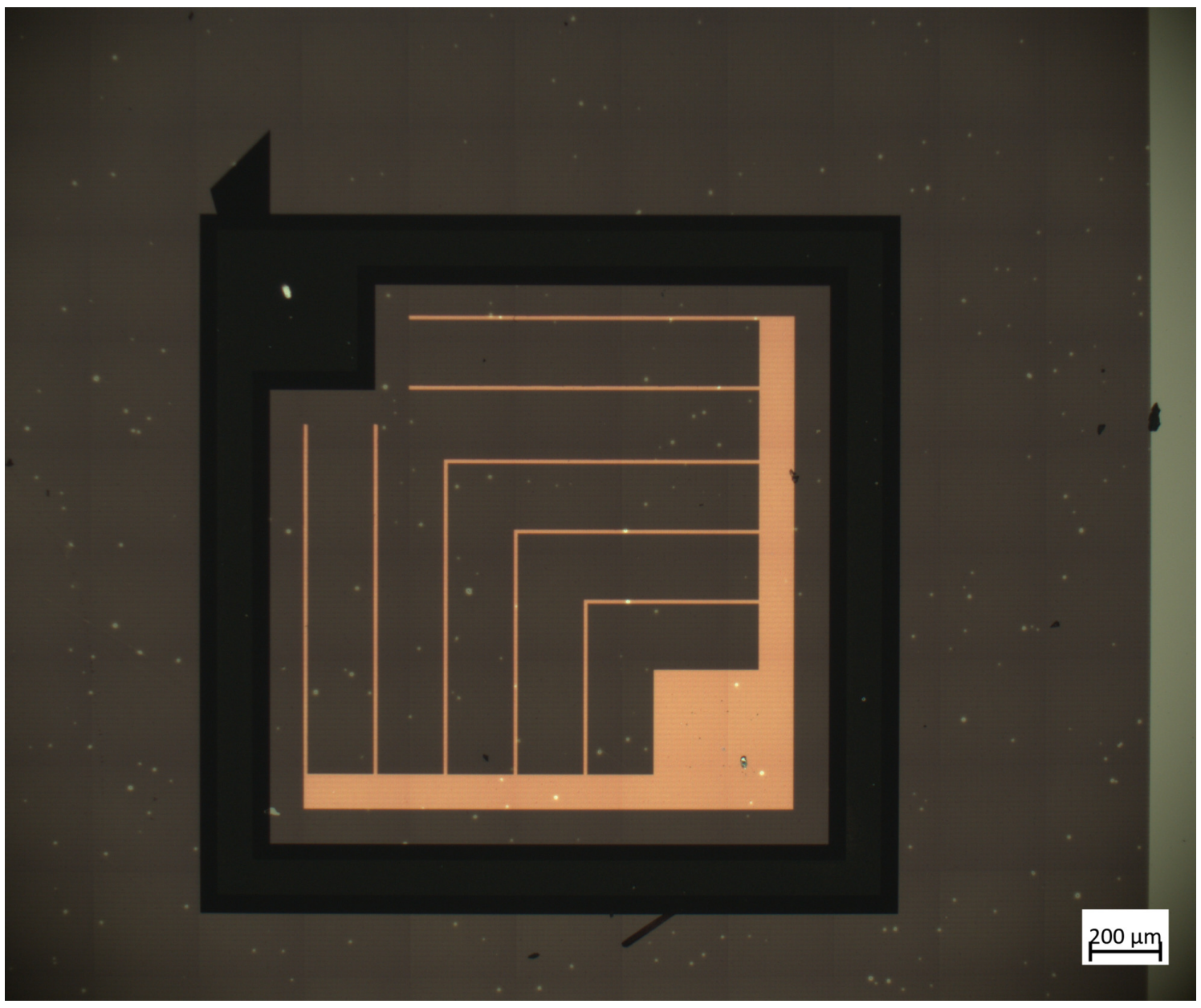

Fig. S11 Device with HUDLE Holes pattern.

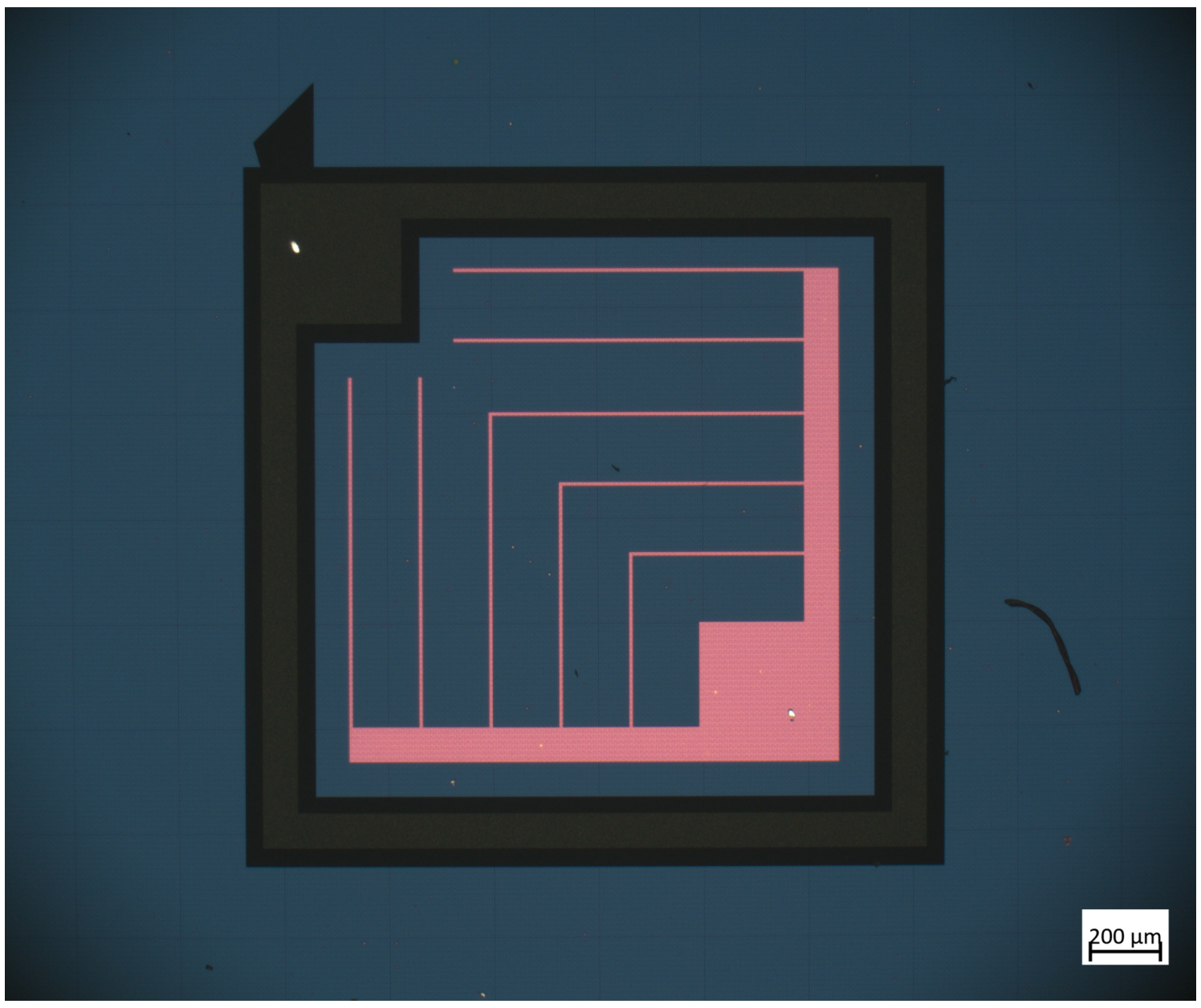

Fig. S12 Device with HUDLE Walls pattern.

References

[1] Yablonovitch, E.: Statistical ray optics. Journal of the Optical Society of America **72**(7), 899 (1982) https://doi.org/10.1364/JOSA.72.000899 . Accessed 2024-11-11

[2] Lee, N., Xue, M., Hong, J., Van De Groep, J., Brongersma, M.L.: Multi-Resonant Mie Resonator Arrays for Broadband Light Trapping in Ultrathin c-Si Solar Cells. Advanced Materials **35**(29), 2210941 (2023) https://doi.org/10.1002/adma.202210941 . Accessed 2024-11-06
